# Bipotential B-neutrophil progenitors are present in human and mouse bone marrow and emerge in the periphery upon stress hematopoiesis

**DOI:** 10.1128/mbio.01599-24

**Published:** 2024-07-16

**Authors:** Shima Shahbaz, Eliana Perez Rosero, Hussain Syed, Mark Hnatiuk, Najmeh Bozorgmehr, Amirhossein Rahmati, Sameera Zia, Jason Plemel, Mohammed Osman, Shokrollah Elahi

**Affiliations:** 1 School of Dentistry, Division of Foundational Sciences, University of Alberta, Edmonton, Canada; 2 Department of Medicine, Division of Gastroenterology, University of Alberta, Edmonton, Canada; 3 Division of Hematology, University of Alberta, Edmonton, Canada; 4 Neuroscience and Mental Health Institute, University of Alberta, Edmonton, Canada; 5 Division of Neurology, Department of Medicine, University of Alberta, Edmonton, Canada; 6 Department of Medical Microbiology and Immunology, University of Alberta, Edmonton, Canada; 7 Department of Medicine, Division of Rheumatology, University of Alberta, Edmonton, Canada; 8 Li Ka Shing Institute of Virology, Faculty of Medicine and Dentistry, University of Alberta, Edmonton, Canada; 9 Cancer Research Institute of Northern Alberta, University of Alberta, Edmonton, Canada; 10 Glycomics Institute of Alberta, University of Alberta, Edmonton, Canada; 11 Women and Children Health Research Institute, University of Alberta, Edmonton, Canada; Griffith University, Gold Coast Campus, Queensland, Australia

**Keywords:** Pro B cell, Pre B cell, myeloid plasticity, hybrid cells, GM-CSF, IL-7

## Abstract

Hematopoiesis is a tightly regulated process that gets skewed toward myelopoiesis. This restrains lymphopoiesis, but the role of lymphocytes in this process is not well defined. To unravel the intricacies of neutrophil responses in COVID-19, we performed bulk RNAseq on neutrophils from healthy controls and COVID-19 patients. Principal component analysis revealed distinguishing neutrophil gene expression alterations in COVID-19 patients. ICU and ward patients displayed substantial transcriptional changes, with ICU patients exhibiting a more pronounced response. Intriguingly, neutrophils from COVID-19 patients, notably ICU patients, exhibited an enrichment of immunoglobulin (Ig) and B cell lineage-associated genes, suggesting potential lineage plasticity. We validated our RNAseq findings in a larger cohort. Moreover, by reanalyzing single-cell RNA sequencing (scRNAseq) data on human bone marrow (BM) granulocytes, we identified the cluster of granulocyte-monocyte progenitors (GMP) enriched with Ig and B cell lineage-associated genes. These cells with lineage plasticity may serve as a resource depending on the host’s needs during severe systemic infection. This distinct B cell subset may play a pivotal role in promoting myelopoiesis in response to infection. The scRNAseq analysis of BM neutrophils in infected mice further supported our observations in humans. Finally, our studies using an animal model of acute infection implicate IL-7/GM-CSF in influencing neutrophil and B cell dynamics. Elevated GM-CSF and reduced IL-7 receptor expression in COVID-19 patients imply altered hematopoiesis favoring myeloid cells over B cells. Our findings provide novel insights into the relationship between the B-neutrophil lineages during severe infection, hinting at potential implications for disease pathogenesis.

**IMPORTANCE:**

This study investigates the dynamics of hematopoiesis in COVID-19, focusing on neutrophil responses. Through RNA sequencing of neutrophils from healthy controls and COVID-19 patients, distinct gene expression alterations are identified, particularly in ICU patients. Notably, neutrophils from COVID-19 patients, especially in the ICU, exhibit enrichment of immunoglobulin and B cell lineage-associated genes, suggesting potential lineage plasticity. Validation in a larger patient cohort and single-cell analysis of bone marrow granulocytes support the presence of granulocyte-monocyte progenitors with B cell lineage-associated genes. The findings propose a link between B-neutrophil lineages during severe infection, implicating a potential role for these cells in altered hematopoiesis favoring myeloid cells over B cells. Elevated GM-CSF and reduced IL-7 receptor expression in stress hematopoiesis suggest cytokine involvement in these dynamics, providing novel insights into disease pathogenesis.

## INTRODUCTION

Hematopoiesis is a tightly regulated process that continuously takes place in the bone marrow niches. This process ensures the orchestrated proliferation of hematopoietic stem cells (HSCs) to give rise to different lineage-committed hematopoietic progenitors under physiological conditions (1, 2). Nevertheless, the hematopoietic system needs to dramatically boost its cellular output to meet the host’s increased demand when the steady condition is disturbed (e.g., infection) (3). The majority of HSCs are quiescent during steady homeostasis, but they rapidly get activated to proliferate and differentiate in response to emergency hematopoiesis (3–5). This is accomplished through regulatory signals within the bone marrow niches from surrounding cells in the form of cell-cell interactions or soluble factors (6–8). Despite multiple differences in the triggering factors, underlying cellular and molecular mechanisms associated with naturally/pathologically occurring emergency hematopoiesis are very similar (9).

One of the major evolutionary factors influencing the hematopoietic system under stress conditions is systemic bacterial infection (3, 10, 11). Upon infection, the most evolutionary survival mechanism aims to enhance the innate immune system via stress myelopoiesis to immediately control invading pathogens (12). This skewing myelopoiesis results in granulopoiesis, monopoiesis, the increased presence of neutrophil progenitors, and erythroid progenitors (1). This is a host defense mechanism to enhance myeloid output to fight off the infection. However, myeloid cells by releasing pro-inflammatory cytokines may promote emergency myelopoiesis (13) at the expense of lymphopenia. In the case of viral infection, most acute viral infections may transiently alter hematopoiesis through the action of different mediators (e.g., type I IFNs and TNF-a) as reported for lymphocytic choriomeningitis virus (LCMV) and influenza infections (13, 14). Moreover, influenza A virus H1N1 promotes emergency megakaryopoiesis and induces pro-coagulant platelets (15). Although SARS-CoV-2 infection is associated with an increase in neutrophil-to-lymphocyte ratio (16, 17), this enhanced myelopoiesis may also perpetuate inflammation in those with the severe form of COVID-19 disease because sustained induced myeloid progenitors in the bone marrow can facilitate the heightened response of these cells and their progeny. Hematopoiesis, once considered irreversible with discrete developmental branches, is now described as a collection of alternative developmental pathways capable of promoting identical progeny (18). Although infection and inflammation have long been known to favor myelopoiesis (e.g., granulopoiesis) over lymphopoiesis, how this process affects B lymphopoiesis in humans is ill-defined (19). For example, it is reported that common lymphoid progenitors (CLP) upon toll-like receptor (TLR) ligation differentiate into dendritic cells (DCs) *in vitro* (19). Hence, redirected differentiation of CLP into DCs may reduce the B cell pool during infection. In line with this observation, it is reported that the reduction in B cells is accompanied by increased production of neutrophils in animal models (20). The reciprocal generation of B cells and neutrophils implies that the progenitors of each group might use a common developmental niche (18). Whether this occurs during infection or chronic inflammatory conditions in humans is less appreciated but presents a significant aspect of clinical management.

In this study, by performing bulk RNA sequencing (RNAseq), we found the presence of unique neutrophils in ICU-admitted COVID-19 patients who were enriched with immunoglobulin and other B cell-associated genes. We further verified our observations in bulk RNAseq data from an independent cohort of COVID-19 patients. Furthermore, using single-cell RNAseq (scRNAseq), we observed the existence of a cluster of granulocytic precursors in the human bone marrow that expresses B cell lineage genes. Moreover, as proof of concept, we confirmed the emergence of myeloid cells from B cells in the bone marrow of mice infected with *E. coli*. Collectively, the diversion of lymphoid progenitors from the B cell lineage to neutrophils may explain how systemic severe infection affects B lymphopoiesis. Therefore, our studies provide novel insights into the emergence of neutrophils from B cell precursors in emergency myelopoiesis.

## MATERIALS AND METHODS

### Animal studies

BALB/c mice were purchased from the Charles River Institute (Morrisville, NC, USA) and were kept and bred under pathogen-free conditions within the animal care facility at the University of Alberta. Bacterial cultures of *E. coli* MC4100 were grown on LB agar plates overnight. The following day, colonies were harvested and resuspended in PBS. Bacterial counts were estimated based on OD600 absorbance, following our established protocols (21, 22). Adult (8–10) weeks male and female mice were infected with *E. coli* MC4100 [intraperitoneally (i.p.), 10^6^ CFU in 100 µL PBS] while control mice received 100 µL of PBS in the same manner (23). The bone marrow (BM) was harvested 48 h later. However, in other studies, at 24 h post-infection, mice were treated i.p. with recombinant IL-7 (5 µg, Stem Cell Technology) and/or GM-CSF (100 µg, Clone MPI-22E9, Bio X cell) for 2 consecutive days before harvesting their BM on day 4 post-infection.

### Human study

**Fig 1:**
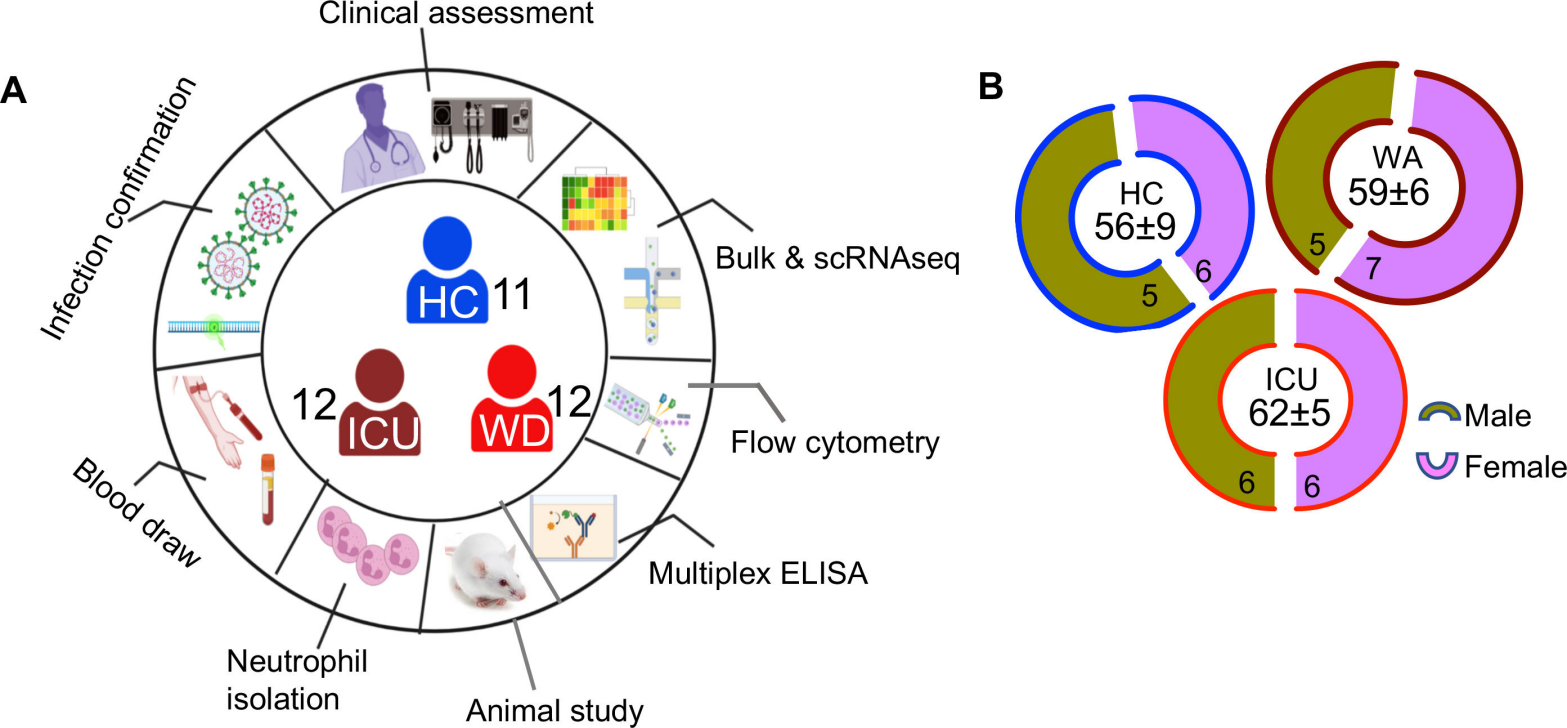
Demographic and the study design. (**A**) Schematic of the study design. Numbers in the center of the diagram indicate participants in each study cohort (HC = healthy controls with no prior-SARS-CoV-2 exposure/vaccination; WD = acute SARS-CoV-2 infected and ward-admitted; ICU = acute SARS-CoV-2 infected and ICD-admitted. Outer ring indicates different studies/assays performed on patients/samples. (**B**) Demographic characteristics for the number of males and females in each cohort are displayed in ring charts. Center values in “age” are median ages in years.

For bulk RNAseq, neutrophils were collected from the fresh blood of 11 HCs, 12 COVID-19 patients in hospital wards, and 12 ICU-admitted patients (Fig. 1A and B). Moreover, we recruited additional 63 hospitalized COVID-19 patients consisting of 22 ICU and 41 ward patients to analyze other related biomarkers. These patients were admitted to different hospitals in Edmonton, Canada. We also used samples collected pre-COVID-19 pandemic from 43 Healthy controls (HCs) who were HIV, hepatitis C virus, and hepatitis B virus seronegative. To prevent confounding factors, we excluded those patients with any co-morbidities.

The diagnosis of SARS-CoV-2 was confirmed by quantitative RT-PCR assay specific for viral RNA-dependent RNA polymerase and envelope transcripts detected using nasopharyngeal swabs or endotracheal aspirates. All of our study subjects were infected with the original Wuhan SARS-CoV-2 strain, and they were SARS-CoV-2 vaccination naive.

### Cell isolation and culture

PBMCs were isolated by gradient separation using Ficoll-Paque PREMIUM (GEU Healthcare, Chicago, USA), and cell pellet comprising neutrophils and red blood cells (RBCs) was subjected to the RBC lysis buffer (0.155M NH4Cl, 10 mM KHCO3, and 0.1 mM EDTA) as reported elsewhere (24–26). Isolated neutrophils with a purity of >97% (Fig. S1A) were washed with PBS and cultured in RPMI 1640 (Sigma-Aldrich) supplemented with 10% FBS (Sigma-Aldrich) and 1% penicillin/streptomycin (Sigma-Aldrich).

BM cells were isolated from the femurs of adult mice (8–10 weeks) by flushing out the marrow to a cell culture Petri dish with a 30-gauge needle attached to a 10 mL syringe using RPMI 1640 media as reported elsewhere (27). For cell culture, isolated bone marrow cells from mice were cultured with recombinant mouse GM-CSF (rGM-CSF 10 /ng, R&D, Cat# 415 mL), IL-6 (10 /ng R&D, Cat# 406 ML-005), and IL-7 (24 ng/mL, Stem Cell Technology) for 48 h. Similarly, human BM aspirates were collected from the femur of 5 adult donors (2 females and 3 males) and subjected to cell isolation and flow cytometry staining according to our protocols (28, 29).

### Flow cytometry analysis

The fluorochrome-conjugated antibodies were purchased from BD Biosciences, Thermo Fisher Scientific, BioLegend, and R&D. The following human antibodies were used for the study: anti-CD15 (HI98, PE), anti-CD16 (3G8, PE Cy7), anti-CD19 (HIB19, FITC & BV421), anti-CD3 (HIT3a, BV605), anti-CD11b (anti-IRCF44, APC, and APC Cy7), anti-CD27(O323, BV540), anti-IgD (IA6-2, BUV395), CD24 (ML5, B515), CD20 (2H7, FITC), and anti-IgM (MHM-88, APC Cy7, and PE-Texas Red). Likewise, a wide range of mouse antibodies were used such as anti-CD19 (1D3/CD19, FITC), anti-B220 (RA3-6B2, BV605), anti-CD11b (M1/70, PerCP-Cy5.5), anti-GM-CSFR (698423, PE), ant-CD114 (680206, APC), anti-CD115 (T38-320, PE), anti-Ly6G (1A8, Alexa 700), anti-Ly6C (HK1.4, PE Cy7), anti-CD25 (PC61.5, Alexa 700), and anti-C-kit (2B8, APC), IL-7R (SB/199, PE-Texas Red), CD27 (LG.3A10, V450), IgM (G53-238, BUV395), CD300a (172224, BV650), CD317 (927, PerCP-Cy5.5), CD79a (F11-172, APC), and CD21 (8D9, PE Cy7). Cell viability was assessed using the LIVE/DEAD kit (Life Technologies). Cell surface staining for humans and mice was performed following our established protocols (30–33). Stained cells were fixed in paraformaldehyde (PFA, 4%), and data were acquired by LSR Fortessa-SORP flow cytometer (BD Biosciences) and analyzed using Flow Jo software (version 10).

### Cytokine and chemokine analysis

The plasma concentration of GM-CSF and IL-7 was measured using the kits from Meso Scale Discovery (MSD) according to the manufacturer’s instructions as reported elsewhere (29, 34).

### Library construction and sequencing

Total RNA was extracted using a Direct-zol RNA MicroPrep kit (Zymo Research) per the manufacturer’s instructions. A total of 100 ng RNA was used to make RNA libraries using TruSeq RNA Library Prep Kit v2 (Illumina, San Diego, CA, USA). Briefly, oligo dTs conjugated to paramagnetic beads were used to pull down polyadenylated mRNAs with non‐polyadenylated transcripts removed by sequential EtOH washes. First‐and second‐strand cDNA was synthesized from chemically fragmented recovered mRNAs. cDNAs were blunted and A‐tailed using T‐A ligation, and finally, 12 cycles of PCR were used to incorporate Illumina adapters containing multiplexing barcodes; the sequencing was performed on a HiSeq 2500 instrument as we reported elsewhere (35). Generated data are publicly available from the SRA portal of NCBI under the Accession Number PRJNA671810.

### Bulk-RNAseq analysis

Pseudo-alignments against the human transcriptome (ensembl Hs_GRCH38) were carried out using Kallisto with bias correction and 100 bootstraps (36). RNAseq data were evaluated at the transcript as well as the gene level where transcript expression was consolidated using tximport (37). Differential expression (DE) analysis was conducted using count data with the DESeq2 R package (R version 4.2.0) (38). Differentially expressed genes demonstrated corrected *P*‐value (*P*_adj_) <0.05 and −1 < log_2_ fold change (FC) > +1. Principal component analysis (PCA), heat map, volcano, box, and UpSet plots were generated using gglot2 with R scripts. Bubble plots were generated using Matplotlib in Python.

### Single-cell-RNAseq (scRNASeq) analysis of human BM

The quality control of single-cell RNAseq (scRNAseq) data obtained from PRJNA482771 was performed in the R statistical environment (v4.1.2) using Seurat v4.0 (https://github.com/satijalab/seurat) (39, 40). The CreateSeuratObject function was used to create Seurat object with the inclusion of the genes expressing in a minimum of 3 cells and cells that expressed a minimum of 200 genes. The subset function was used further to remove cells with > 3,000 gene counts (doublets or multiplets and dead cells) and cells with high percentages (> 10%) of mitochondrial genes. We also subsetted specific groups from a single file to look at specific cell subsets. The normalization of data was performed using the SCTransform function (highly variable features = 3,000) according to the binomial regression model (41). RunPCA, FindNeighbours, and FindClusters functions were used for dimensionality reduction with a retention of 25 PCs, as determined by a PCA elbow plot. The clustering of the data set was performed by the FindCluster using varied resolution parameters (0–1) with successive resolutions separated by −.1 (42). A tree was created with all clusters at all resolutions using the clustree function, and a resolution of 0.6 was selected for our clustering. SeuratDisk was used to convert the data set to an h5ad file that was further loaded into a Python environment (Python 3.8.3) using Scanpy (v1.6.0) (43). We further refined the cells to reach a 95% self-projection accuracy using the Single Cell Clustering Assessment Framework (SCCAF) package (v0.0.10, https://github.com/SCCAF/sccaf) (44), and the final clustering iteration was used to make a UMAP. Annotation of the clusters was performed using SingleR package (45).

### Hierarchical clustering of differentially expressed genes

The differentially expressed genes (DEGs) in each cell subpopulation were calculated using the FindAllMarkers function in Seurat (min. pct  =  0.25, logFCthreshold  =  0.25, *P*  <  0.05). After the calculation of the euclidean distance between the DEGs using the dist function, the DEGs were further clustered using Ward’s methods (ward. D2) in the hclust function of the stats R package (v4.1.2), and the average gene expression values were plotted in Seurat using the DoHeatmap function. The plot_density function of the Nebulosa package (v1.4.0) was used for the visualization of the genes through density plots (46).

### scRNA-seq analysis of the BM in mice

The scRNAseq of neutrophil populations under homeostasis and bacterial infection is publicly available at https://www.ncbi.nlm.nih.gov/geo/query/acc.cgi?acc=GSE137539 (47).

We obtained scRNAseq counts from the GEO database in the form of a raw expression matrix of unique molecular identifier (UMI) counts, representing the number of unique RNA molecules detected for each gene in each cell group. UMI counts from each data set were filtered based on the following criteria: cells with more than 200 UMI counts, more than 200 features, less than 4,000 features, and less than 5% mitochondrial gene expression. Subsequently, we normalized and log-transformed the data using the *NormalizeData* and *ScaleData* functions with a 10,000-scale factor. To integrate the data sets, we utilized the *harmony* package for batch correction and scRNAseq data integration. We then performed a combined PCA, computing 50 principal components. Based on the elbow plot, we selected the first 30 principal components for Uniform Manifold Approximation and Projection (UMAP) analysis for dimensionality reduction. Next, we clustered cells with a resolution of 0.5. Finally, we annotated cell clusters using the *singleR* package and the *Tabula Muris* 10× data set for bone marrow.

### Statistical analysis

Statistical analysis was performed using GraphPad Prism 9 (GraphPad Software, Inc.). Mann-Whitney *U* test was used to compare nonpaired data sets. When more than two groups were compared, a one-way analysis of variance (ANOVA) followed by Tukey’s test was used. Data were presented as means and standard deviations (mean ± SEM).

## RESULTS

### Differential transcriptional profile of neutrophils from COVID-19 patients

To determine differences between the transcriptional profile of neutrophils in HCs, ward-, and ICU-admitted COVID-19 patients, we conducted bulk RNAseq on total RNA extracted from neutrophils of 11 HCs, 12 ward-admitted, and 12 ICU-admitted COVID-19 patients who were age-sex-matched (Fig. 1A and B). PCA based on Euclidean distances separated neutrophils of HCs from ICU-admitted COVID-19 patients in a two‐dimensional plot except for one study subject (Fig. S1B). Similarly, PCA separated neutrophils of HCs from ward-admitted patients, with the exception of one patient/group (Fig. S1C). However, neutrophils from SARS-CoV-2-infected groups either admitted to the ICU or ward exhibited a mixed transcriptional profile with three ICU and four ward patients showing similar transcriptional pattern to the other group (Fig. S1D). More specifically, compared with HCs, 5,105 and 2,783 transcripts were found to significantly up- and down-regulated in neutrophils from ICU-admitted patients, respectively (Fig. S1E, S2A, and B). When neutrophils from HCs were compared with ward-admitted subjects, the observed transcriptional changes were less dramatic, only 3,035 and 904 transcripts were significantly up- and down-regulated, respectively (Fig. S1F, S2A, and C). Finally, the comparison between ward and ICU-admitted COVID-19 patients revealed the significant upregulation and downregulation of 190 and 221 transcripts, respectively (Fig. S1G, S2A, and D).

Next, we identified the five most up- and down-regulated transcripts in each set of comparisons. When we compared HCs with the ICU group, the most upregulated transcripts were TNXB, MGAM, WTAP, LILRB3, and CANX, whereas the most downregulated transcripts were DNM2, RABGAP1L, AMPD2, KANSL1, and PPP2R5A (Fig. S2E). The Tenascin-X (TN-X) B is a member of the Tenascin family of proteins that modulate cell adhesion (48). Its high expression in neutrophils of ICU patients might contribute to enhancing chemotaxis. The maltose-glucoamylase (MGAM) is mainly expressed in the intestinal brush border and is involved in terminal starch product digestion (49); however, its role in the immune system and neutrophils remains to be determined. The third and fourth upregulated transcripts were WTAP and LILRB3, which encode for the Wilms Tumor 1-associating protein (WTAP) and Leukocyte Ig-like Receptor B3 (LILRB3), respectively. While WTAP positively regulates the IFN-I signal to enhance antiviral response (50), LILRB3 modulates the maturation and activation of neutrophils (51). In addition, LILRB3 enhances the induction of myeloid suppressor cells (52). Finally, calnexin (CANX) is involved in the formation of phagosome membranes and endoplasmic reticulum (ER)-mediated phagocytosis (53). The most downregulated transcript in neutrophils of ICU patients was dynamin 2 (DNM2), whose deficiency is indicative of disrupted bone marrow hemostasis and expansion of progenitor and hematopoietic stem cells (54). RAB GTPase Activating Protein 1 Like (RABGAP1L) has an anti-inflammatory property (55), and AMPD2 encodes for Adenosine Monophosphate Deaminase 2 exhibits an anti-inflammatory function through modulation of ATP-adenosine balance (56). Finally, KANSL1 and PPP2R5A encode K (lysine) acetyltransferase 9 (KAT8) Regulatory NSL Complex Subunit 1 and Protein Phosphatase 2 Regulatory Subunit B Alpha, respectively. KAT8 regulates the expression of antioxidant genes, and its deficiency is associated with an increase in reactive oxygen species (ROS) production (57). Of note, the downregulation of PPP2A, which augments the levels of phosphorylated p38 MAPK and caspase 3, with subsequent decreased activity of caspase 3 and neutrophil apoptosis, likely enhances neutrophil survival (58). Overall, these analyses suggest the expansion of neutrophil progenitors possessing a higher activation and survival phenotype in ICU-admitted patients.

Comparison of neutrophils from HCs and ward patients revealed RNF149, WTAP, OTOF, MBD3L1, and MDM4 as the most upregulated and DICER1, BRD2, TRIM39, SERPINA1, and DDAH2 as the most downregulated transcripts (Fig. S2F). The ring finger protein 149 (RNF149) binds to BRAF, one of the members of the RAF family, and is involved in cell growth inhibition (59). The potential role of the WATP transcript was discussed above (Fig. S2E). The other highly upregulated transcript was OTOF, which encodes the transmembrane protein Otoferlin and is induced upon IFN-α stimulation (60). Although OTOF prevents HIV-1 infection in macrophages and dendritic cells (60), its role in SARS-CoV-2 infection is unclear. The other two highly upregulated transcripts were MBD3L1 (Methyl-CpG Binding Domain Protein 3 Like 1) and MDM4 encode (Mouse Double Minute four protein). MBD3L1 is a transcriptional repressor with gene silencing capability via binding to both unmethylated and methylated DNA (61), which promotes cell proliferation through negative regulation of p53 gene (62).

In analyzing downregulated transcripts in ward patients, we found that Dicer1 was the most downregulated transcript, which is involved in monocytic differentiation and neutrophil maturation (63). The second most downregulated transcript, Bromodomain Containing 2 (BRD2), may block SARS-CoV-2 infection by downregulating ACE2 expression (64). The next most downregulated transcripts TRIM39 and SERPINA1 encode Tripartite Motif Containing 39 and alpha-1 antitrypsin (AAT) protein, respectively. Trim39 inhibits the ubiquitination and degradation of p21 (65). As a result, decreased Trim39 expression suggests a higher proliferative capacity of neutrophils in these patients. On the other hand, the AAT protein protects human tissues from the destructive damage of neutrophilic enzyme elastase (66), and its downregulation might be associated with pulmonary interstitial emphysema observed in COVID-19 patients (67). Finally, reduction in Dimethylarginine Dimethylaminohydrolase 2 protein (DDAH2) expression may reduce NO synthesis and signaling (68). Taken together, these analyses reveal that neutrophils in ward patients exhibit an inflammatory phenotype compared with their counterparts in HCs.

Finally, we compared transcripts in neutrophils between COVID-19 patients. As shown in Fig. S2G, we found FLOT1, C6ORF48, APOBEC3A, ANKRD11, and IKBKG as the most upregulated transcripts while OR2H1, TRAF5, DMXL2, TMEM50B, and DNMT1 constituted the most downregulated transcripts in this group. Flotillin-1 (FLOT1) contributes to the chemotactic function of neutrophils (69), but C6ORF48 (the chromosome six opening reading frame 48) is involved in cell cytoskeleton formation with increased expression in patients infected with parvovirus B19 (70). APOBEC3A contributes to the phagocytic function of neutrophils by deaminating cytidines to uridines in foreign double-stranded DNA (71). Other upregulated transcripts were Ankyrin Repeat Domain Containing 11 (ANKRD11) and Inhibitor of Nuclear Factor Kappa B Kinase Regulatory (IKBKG) Subunit Gamma proteinase. The upregulation of ANKRD11 in neutrophils of COVID-19 patients following dexamethasone treatment has already been noted (72). IKBKG encodes nuclear factor-κB (NF-κB) essential modulator (NEMO), which has an important regulatory role in the IκB kinase protein complex through the activation of NF-κB, a critical transcriptional regulator in neutrophils (73).

On the other hand, Olfactory Receptor Family 2 Subfamily H Member 1 (OR2H1), the most downregulated gene in neutrophils of ICU patients may contribute to the chemotactic function of neutrophils (74). The downregulation of TNF Receptor Associated Factor 5 (TRAF5) is linked to increased adhesion of inflammatory cells to endothelial cells (75). The next downregulated transcript DMX-like 2 proteins (DMXL2) is highly expressed in SiglecF^HI^ neutrophils or late-stage neutrophils (76). Thus, decreased expression of DMXL2 in neutrophils from ICU patients suggests the existence of newly generated neutrophils in these patients. TMEM50B and DNMT1, the last downregulated genes, encode Transmembrane Protein 50B and DNA methyltransferase 1, respectively. While the role of Transmembrane Protein 50B in neutrophils is unknown, the high DNA methyltransferase activity is associated with greater expression of myeloperoxidase (MPO) and proteinase 3 (PRTN3), two important enzymes in neutrophil granule (77). Therefore, our analyses of transcripts revealed that COVID-19 patients have a greater proportion of newly generated neutrophils with enhanced inflammatory properties.

### Higher expression of genes associated with chemotaxis, phagocytosis, and cytotoxicity in neutrophils of COVID-19 patients

**Fig 2:**
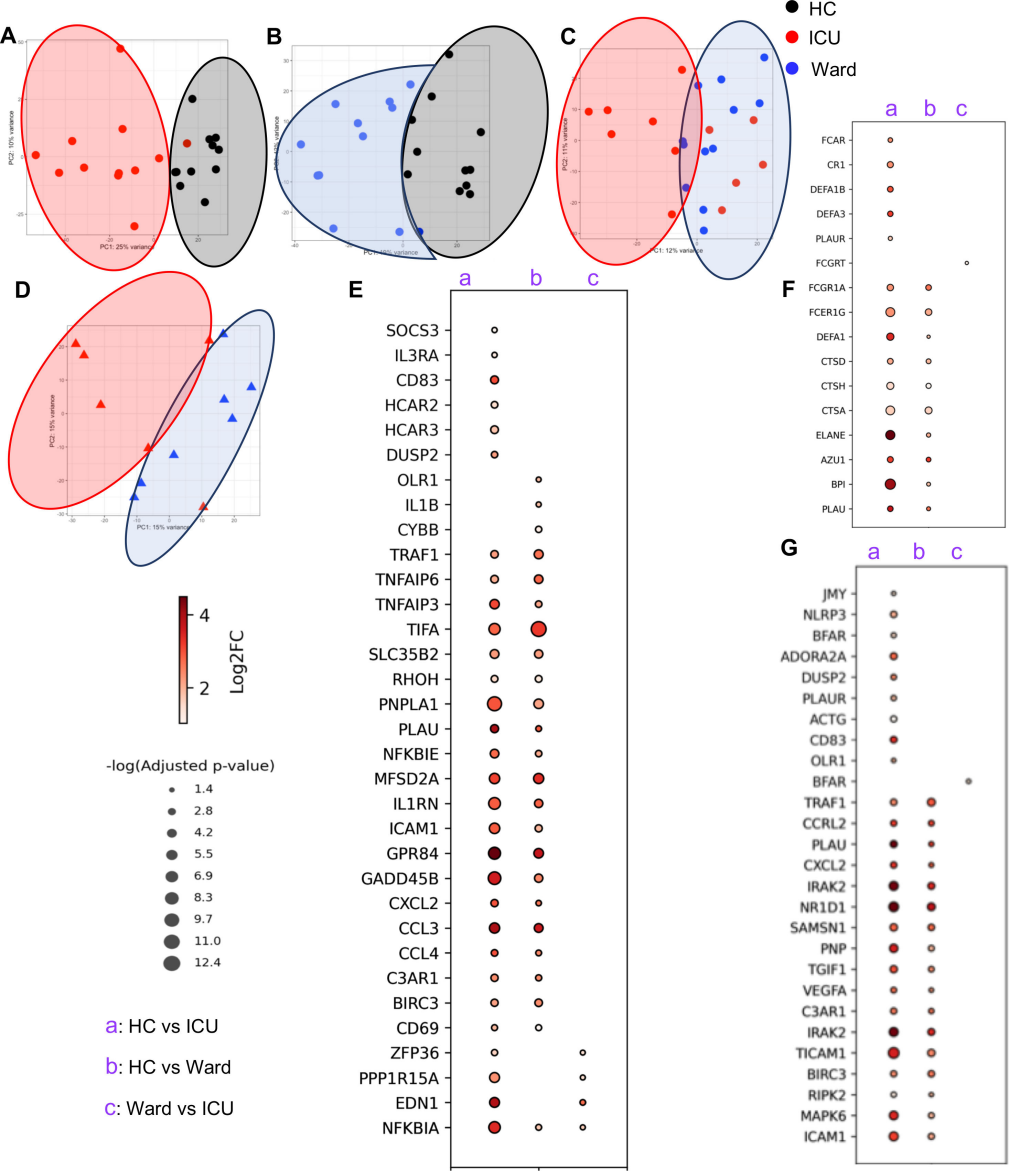
Neutrophils from COVID-19 patients are enriched with genes contributing to enhanced phagocytic, chemotactic, and cytotoxic potentials. (**A**) PCA on the euclidian distances of the genes between neutrophils of HC vs. ICU-admitted, (**B**) HC vs. ward-admitted, (**C**) ICU vs. ward-admitted, and (**D**) ICU-admitted males vs. ward-admitted male COVID-19 patients. Bubble plots showing the differential expression of genes associated with (**E**) chemotaxis, (**F**) phagocytosis, and (**G**) cytotoxic function of neutrophils in our three different cohorts. Dot size represents the −log (p_adj_) >1.3, and the colors represent the fold change of the genes in each comparison.

Next, the transcript level expression was consolidated to gene level expression using tximport to identify pathways of relevance. Similar to transcripts, PCA revealed that the gene expression pattern of neutrophils in HCs was different from both groups of COVID-19 patients (Fig. 2A and B). However, this was not the case when we compared ward and ICU patients. As shown in Fig. 2C, four ICU patients deployed a similar gene expression profile to ward patients. Further analysis indicated that compared with HCs, neutrophils from ICU and ward patients had 2,677 and 1,480 upregulated genes and 1,714 and 390 downregulated genes, respectively (Fig. S3A and B). Moreover, neutrophils from ICU patients exhibited 370 and 587 up- and down-regulated genes compared with ward patients (Fig. S3C).

Given the influence of sex as a biological factor in neutrophil effector functions (78, 79), we considered sex in our analysis. These analyses revealed that neutrophils from ICU-admitted male patients were mostly separated from those males admitted to hospital wards (Fig. 2D), which showed 495 and 311 up- and down-regulated genes, respectively (Fig. S3D). However, this was not the case for neutrophils from female patients as they showed a small number of differentially expressed genes between ICD-admitted and ward patients (Fig. S3E and F).

Next, we analyzed the functionality of neutrophils based on their gene expression profile. These analyses revealed a significantly higher expression of several genes associated with chemotaxis in neutrophils of ICU and ward patients compared with HCs (Fig. 2E). However, the difference between neutrophils from ICU and ward patients was limited to only four chemotaxis-associated genes (Fig. 2E). Next, we compared the phagocytic and killing capacity of neutrophils in our three different cohorts. Similar to chemotaxis, we observed a higher expression of genes associated with both phagocytosis and cytotoxicity of neutrophils in ICU and ward patients compared with HCs, with fewer differentially expressed genes in the later comparison (Fig. 2F and G). However, when a similar comparison was performed between neutrophils from ICU and ward patients, we noted only one differentially expressed gene (Fig. 2F and G). These findings, in agreement with other reports (80), suggest that neutrophils from COVID-19 patients possess greater chemotactic, phagocytic, and cytotoxic capabilities compared with HCs, which were more prominent in neutrophils from ICU-admitted patients.

### Enrichment of B cell linage genes in neutrophils of COVID-19 patients

**Fig 3:**
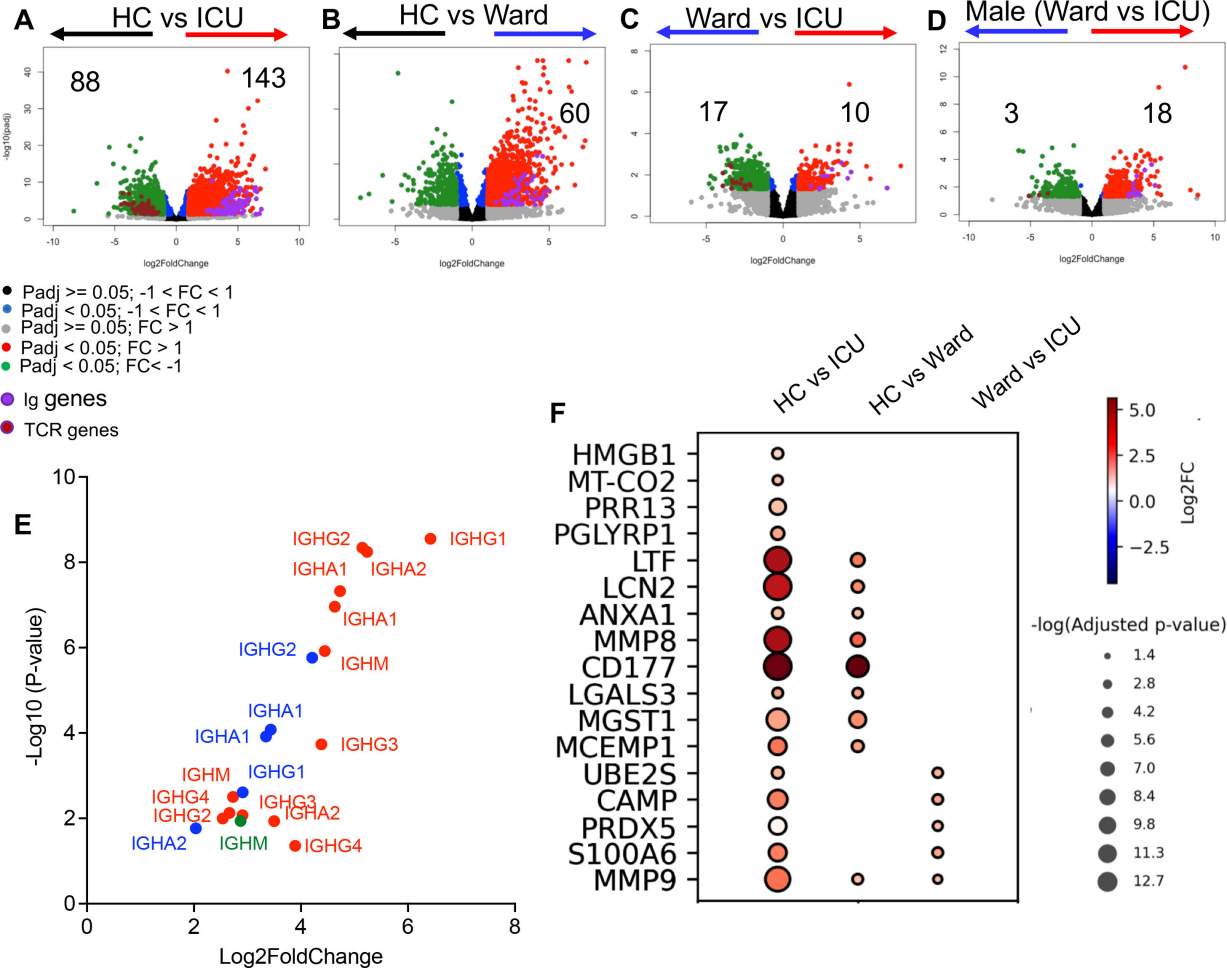
Neutrophils from COVID-19 patients are enriched with Igs and B cell-associated genes. Volcano plots depicting the number and fold change (FC) of differentially expressed Ig and TCR genes in neutrophils of (**A**) HCs vs ICU-admitted, (**B**) HCs vs ward-admitted, (**C**) ward vs ICU-admitted groups, and (**D**) male patients in ICU vs ward. Purple dots represent Ig, and brown dots represent TCR genes. (**E**) Volcano plot showing the number and FC of the genes of different classes of Igs as shown by the type of heavy chains in our three different cohorts. (**F**) Bubble plot of differentially expressed genes associated with immature neutrophil phenotypes presented in three groups comparison.

Considering that SARS-CoV-2 infection is associated with an increase in neutrophils-to-lymphocyte ratio (9, 72), we hypothesized that stress hematopoiesis in COVID-19 patients may promote the conversion of early B cell progenitors to neutrophils as reported in an animal infection model (18). In agreement with this hypothesis, we observed a robust upregulation of immunoglobulin (Ig) genes (*n* = 143) in neutrophils from ICU patients compared with HCs (Fig. 3A). However, this was not the case for T cell receptor (TCR)-associated genes in neutrophils of ICU patients. Indeed, we noted 88 genes associated with TCR were significantly downregulated in neutrophils from ICU patients compared with HCs (Fig. 3A). A similar comparison between neutrophils from ward-admitted patients with HCs revealed the enrichment of 60 Ig genes without any changes in TCR-associated genes (Fig. 3B). However, the difference in the expression of Ig-associated genes between neutrophils of ICU and ward-admitted patients was restricted to 10 genes (Fig. 3C). Moreover, we found that neutrophils from male ICU patients admitted exhibited the upregulation of 18 Ig genes and downregulation of three TCR-related genes (Fig. 3D). Interestingly, we did not observe any significant difference in the expression of Ig-associated genes in neutrophils of female patients (Fig. S3F). Our further analysis revealed that the upregulated Ig genes in neutrophils from COVID-19 patients belonged to different classes of Igs (Fig. 3E). We found that neutrophils in COVID-19 patients exhibit upregulation of Ig genes, with the highest numbers of upregulated genes seen in patients admitted to ICU. In contrast, there was a downregulation of TCR genes in neutrophils from COVID-19 patients.

**Fig 4:**
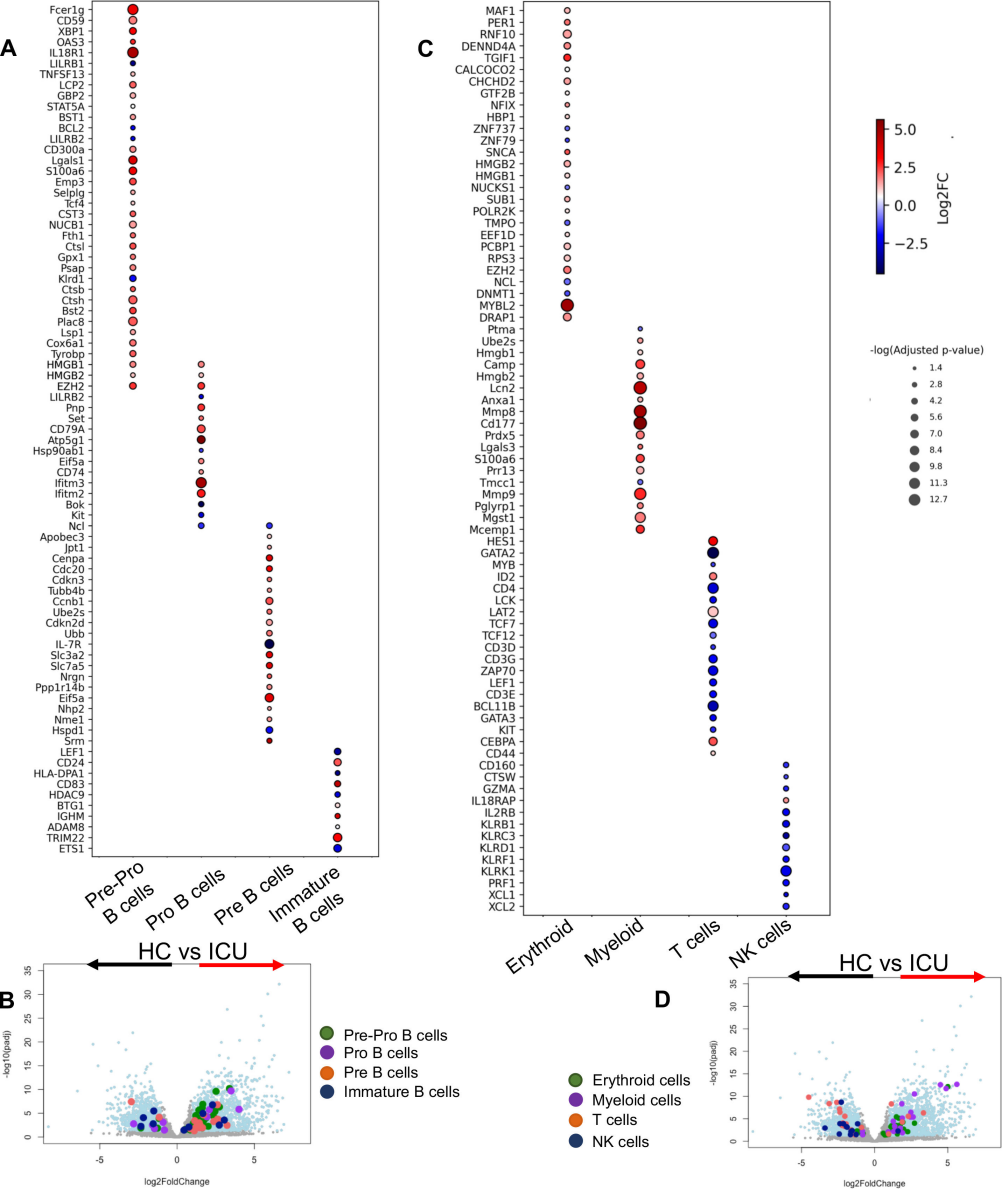
Neutrophils from COVID-19 patients possess B cell-associated genes. (**A**) Bubble plot of differentially expressed genes associated with different stages of B cell development in neutrophils of HC vs ICU patients. (**B**) Volcano plot of the number and FC of genes associated with different stages of B cell development in neutrophils of HC vs ICU patients. (**C**) Bubble plot of differentially expressed genes associated with different types of immune cells in neutrophils of HC vs ICU patients. (**D**) Volcano plot of the number and FC of genes associated with different immune cells in neutrophils of HC vs ICU patients.

Also, neutrophils from COVID-19 patients were enriched with a wide range of genes associated with an immature neutrophil phenotype (Fig. 3F), which agrees with the concept of enhanced myelopoiesis in the context of SARS-CoV-2 infection (81). Given the high purity of our isolated neutrophils (> 97%) and a lack of mature B cell contamination (Fig. S1), the enriched Ig gene signature implies the presence of B cell lineage cells in the neutrophils of COVID-19 patients. This concept was further supported by the enrichment of genes associated with different stages of B cell development (e.g., pre-pro, pro-B, pre-B, and immature B cells) in neutrophils from ICU-admitted patients (Fig. 4A and B). Furthermore, we observed the upregulation of genes associated with erythroid and myeloid lineages but the downregulation of genes related to T and Natural killer cell (NK cell) lineages in neutrophils of ICU patients (Fig. 4C and D). Our analysis of neutrophils from ward-admitted patients revealed a similar pattern, however, with a lower intensity of expression. These observations confirm the enrichment of genes associated with B cell, myeloid, and erythroid lineages in neutrophils from COVID-19 patients.

### Verification of enriched B cell lineage genes in neutrophils of COVID-19 patients in a validating cohort

**Fig 5:**
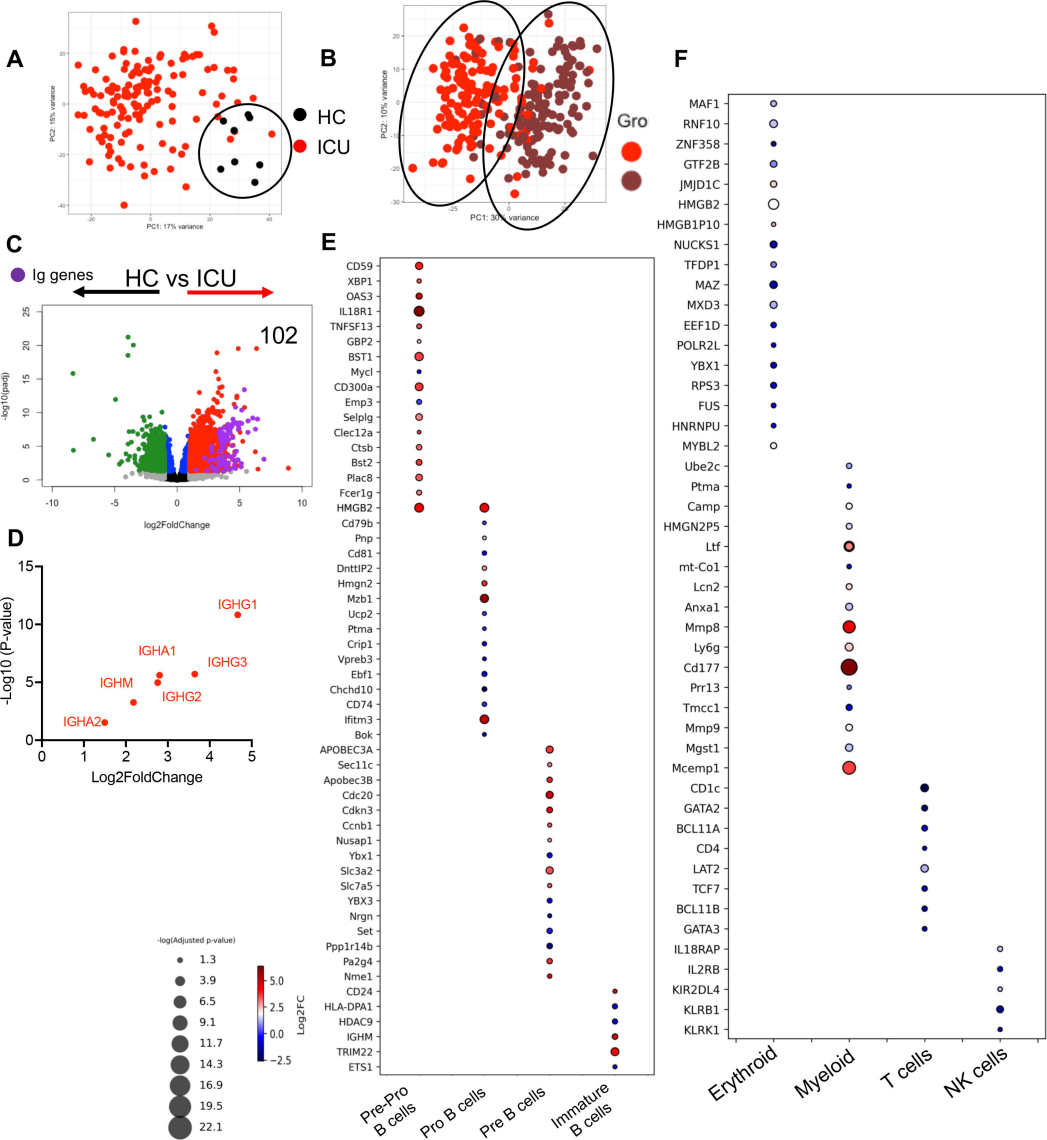
Enrichment of B cell-associated genes in the neutrophils of the validating cohort. PCA on the Euclidian distances of the genes between neutrophils of (**A**) HCs and COVID-19 patients at the time of ICU admission and (**B**) COVID-19 patients at the time of admission and 7 days after admission to the ICU. (**C**) Volcano plot depicting the number and FC of differentially expressed Ig genes in neutrophils of HCs vs ICU patients. Purple dots represent Ig genes. (**D**) Volcano plot showing the number and FC of the genes of different classes of Igs as shown by the type of heavy chains in COVID-19 patients. (**E**) Bubble plot of differentially expressed genes associated with different stages of B cell development in neutrophils of HC vs ICU patients. (**F**) Bubble plot of differentially expressed genes associated with different types of immune cells in neutrophils of HC vs ICU patients.

To verify our observations, we conducted a similar analysis in another cohort of COVID-19 patients admitted to ICU at the Massachusetts General Hospital. This group subjected neutrophils from 388 COVID-19 patients at different time points post-admission to ICU compared with eight HCs to bulk RNAseq (82). To mimic our sample collection time, we considered samples obtained at the time of admission and 7 days later (*n* = 136) for our analysis. Similar to our cohort, PCA clearly separated neutrophils from HCs and ICU patients when collected at the time of admission to the ICU (Fig. 5A). Interestingly, neutrophils isolated 7 days post patients’ admission to ICU were mainly segregated together and separated from their counterparts isolated at the admission time except for a few samples that deployed a transcriptional profile to the other group (Fig. 5B). In agreement with our cohort, we noted the enrichment of 102 genes from different classes of Igs in neutrophils of ICU-admitted patients (Fig. 5C and D). Moreover, we noted the enrichment of genes associated with different stages of B cell development in neutrophils from this cohort (Fig. 5E), which provides additional support to our hypothesis. Consistent with our observations in the discovery cohort (Fig. 4C), we observed the upregulation of myeloid but downregulation of T and NK cell signature genes in neutrophils of this validating cohort (Fig. 5F). However, the enriched erythroid signature was not pronounced in this cohort (Fig. 5F). Hence, these analyses validated our initial observation of the presence of B cell signature genes in neutrophils from COVID-19 patients.

### Granulocytes from human bone marrow possess B cell genes signature

Considering the existence of a hybrid cell phenotype (B-myeloid cell) in the peripheral blood of COVID-19 patients, we decided to determine whether such a cell hybrid phenomenon can be observed under physiological conditions in human BM. To test this hypothesis, we re-analyzed a scRNAseq data set that was performed on seven different subpopulations of human BM cells (83). We analyzed the gene expression profile of granulocyte–monocyte progenitors (GMP), Pre-B lymphocytes/natural killer cells (Pre-B/NK), megakaryocyte-erythroid progenitors (MEP), and common myeloid progenitors (CMP) subpopulations. Low-quality cells were discarded (Fig. S3G) and unsupervised clustering was performed using the Louvain algorithm (39). We further used Single-Cell Clustering Assessment Framework (SCCAF), which accurately identifies a cell identity using a machine-learning approach to refine clustering (44).

**Fig 6:**
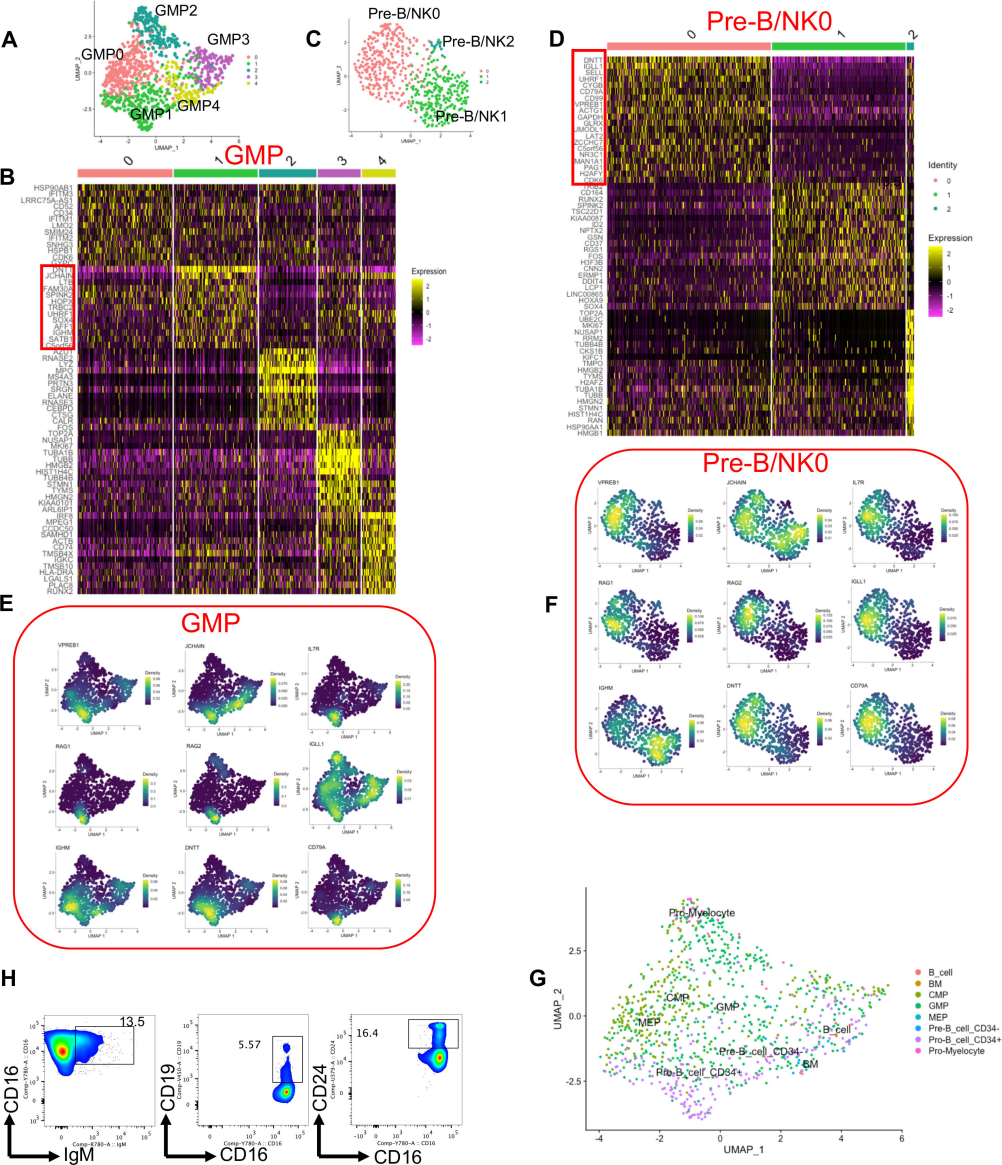
High expression of B cell signature genes in BM granulocytic progenitors. (**A**) UMAP plot of GMP clusters. (**B**) The top 13 upregulated genes in every GMP cluster. (**C**) UMAP plot of Pre-B/NK0 clusters. (**D**) The top 20 upregulated genes in every B/NK0 cluster. Density plots depicting the level of expression of B-cell lineage-associated genes in (**E**) GMP and (**F**) Pre-B/NK0 subpopulations. (**G**) Annotation of GMP clusters using SingleR. (**H**) Representative flow cytometry plots of percentages of neutrophil expression IgM, CD19, and CD24 in a human BM sample.

We first projected GMP and Pre-B/NK cells onto a Uniform Manifold Approximation and Projection (UMAP) to visualize clustering. We identified 5 and 3 different clusters in GMP and Pre-B/NK subpopulations, respectively (Fig. 6A through D). Interestingly, we found that cluster 1 of GMPs possesses a high expression level of B cell lineage-associated genes (Fig. 6B), which belongs to cluster 0 of the Pre-B/NK cell subpopulation (Fig. 6D). In particular, we found that B cell-lineage-associated genes such as *VPERB1, JCHAIN, IL7R, RAG1, RAG2, IGLL1, IGHM, DNTT*, and *CD79A* were highly enriched in GMPs (Fig. 6E). As expected, the same set of genes were abundant in cluster 0 of Pre-B/NK cells (Fig. 6F). We cross-annotated clustered GMPs using a singleR package, which labeled GMP1 cluster as Pre- and Pro-B cells (Fig. 6G). To investigate whether other BM progenitors also express B cell lineage-associated genes, we assessed the expression level of these genes in CMP and MEP subpopulations. UMAP clustering of CMP and MEP cells identified five and six clusters in MCP and MEP cells, respectively (Fig. S4A through D). Although we observed the expression of *VPERB1, IL7R, RAG1, RAG2, IGLL1, IGHM*, and *CD79A* in the CMP subpopulation (Fig. S4E) and *RAG1, RAG2, IGLL1, IGHM*, and *CD79A* in MEPs (Fig. S4F), these genes were spread among different clusters without a unique clustering to mimic the expression pattern of B cells lineage signature genes. Moreover, we investigated the presence of proteins associated with B cells in BM neutrophils from humans. Despite the small sample size (*n* = 5), we consistently observed the presence of IgM, CD19, and CD24 in neutrophils from the human BM (Fig. 6H; Fig. S5A and B). CD24 is detected in immature B cells, precedes the expression of CD19, and takes place concurrently with the commitment of progenitors to B cell lineage (84, 85). Likewise, the presence of CD19 in the absence of CD20 expression (Fig. S1A) suggests the existence of Pro Pre-B cells (86) in the BM-derived neutrophils (Fig. 6H; Fig. S5B). Collectively, these results show the existence of a cluster of hybrid cells in BM progenitors co-expressing the gene signature of both GMP and B cell lineages.

### Expansion of CD11b+B cells in the BM of infected mice during emergency myelopoiesis

The classical view of B cell development is challenged by recent studies that have provided concise evidence that acute and chronic inflammation may impact B cell development in the BM and periphery (87, 88). During development, B cells are derived from HSCs in a process of repressing traits of other immune cell lineages (89). This suggests that during severe infection, such a regulatory process may give rise to myeloid cells.

**Fig 7:**
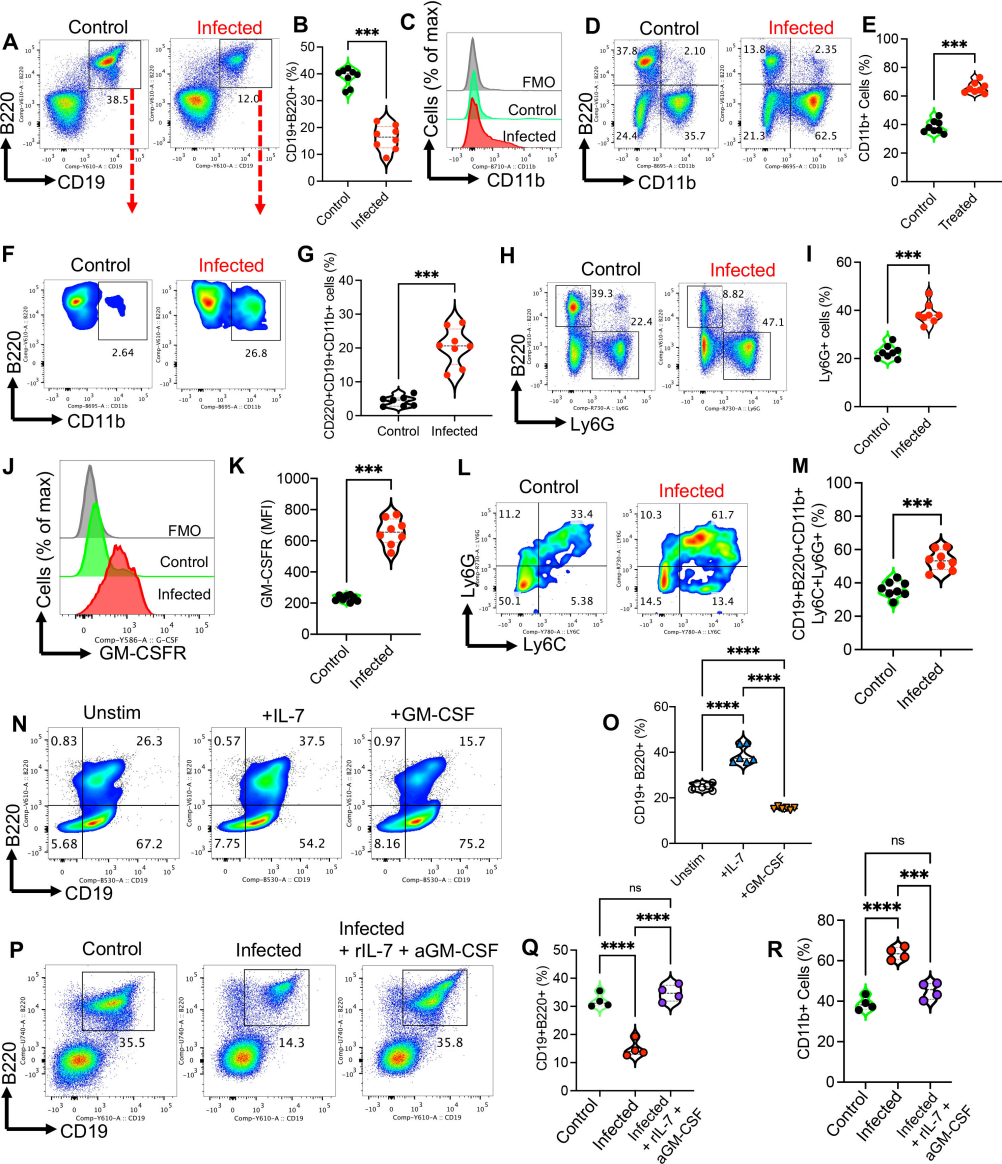
Systemic infection expands myeloid cells within B cell subset in the BM of mice. (**A**) Representative flow cytometry plots, and (**B**) cumulative data of percentages of CD19 + B220+ cells in control and infected mice with *E. coli* (i.p., 10^6^) 48 hr later. (**C**) Representative histogram showing the mean fluorescence intensity (MFI) of CD11b among CD19 + B220+ cells in a control and an infected mouse. (**D**) Representative and (**E**) cumulative data of percentages of CD11b+ cells in the BM of control vs infected mice. (**F**) Representative flow cytometry plots, and (**G**) cumulative data of percentages of CD11b+ cells within B220 + CD19+ cells in control and infected mice. (**H**) Representative flow cytometry plots, and (**I**) cumulative data of percentages of Ly6G+ cells in control and infected mice. (**J**) Representative histogram plot and (**K**) cumulative data of MFI for GM-CSF-receptor (**R**) in CD19 + B220+ cells in the BM of control and infected mice. (**L**) Representative plots and (**M**) cumulative data of percentages of Ly6C + Ly6G + cells among CD19 + B220+ cells obtained from the BM of control and infected mice. (**N**) Representative flow cytometry plots, and (**O**) cumulative data of percentages of CD19 + B220+ cells in BM cells obtained from either control and infected mice in the absence of cytokines (unstim) or treated with IL-7 (24 ng/mL) and rGM-CSF (10 ng/mL) for 24 hr *in vitro*. (**P**) Representative plots and (**Q**) cumulative data showing the percentages of B220 + CD19+ cells in the BM of control, infected, and infected plus treated with rIL-7 (5 µg/mouse) and anti-GM-CSF (100 µg/mouse) for 2 consecutive days. (**R**) Cumulative data of the percentages of CD11b+ cells in the BM of control, infected, and infected plus treated with rIL-7 (5 µg/mouse) and anti-GM-CSF (100 µg/mouse) for 2 consecutive days. Each dot represents data from an animal either control or infected. *P* values were calculated using two-tailed, Mann-Whitney *t* test (**B, E, G, I, K, M**). One-way ANOVA (**O, Q, R**). ***, *P* < 0.001, and ****P < 0.0001. Unstimulated (unstim), Fluorescence minus one (FMO), not significant (ns).

The suppression of lymphopoiesis during emergency myelopoiesis (e.g., infection) is considered a smart strategy to supply abundant innate immune cells as the first line of defense (87). Therefore, to understand the potential mechanism underlying this phenomenon, we tested this concept in an acute systemic infection model (e.g., *E. coli*) in adult BALB/c mice. We found a significant reduction in the proportion of B220 + CD19+ B cells (Fig. 7A and B) but an increase in the intensity and frequency of CD11b+ cells, a pan-myeloid marker, in the BM of mice post-infection (Fig. 7C through E). While B220 + CD19+ cells were diminished, we found a significant increase in the frequency of CD11b cells in the BM of infected mice (Fig. 7F and G). Similarly, the proportion of Ly6G cells was increased in the BM of infected mice compared with the control group (Fig. 7H and I). CD19+ B cells are composed of mature (B220^high^IgM^+^), immature (B220^low^IgM^+^), and B cell precursors (B220^+^IgM^−^) (Fig. S5C). We further found a reduction in the proportion of pre-B cells among CD19 + B220+ B cells in infected mice (Fig. S5D and E). Upon infection, the frequency of B cell precursors dramatically reduced, and the number of mature B cells conversely increased (Fig. S5C and E). This implies the differentiation of B cell precursors into mature B cells upon emergency myelopoiesis. Given the migration of immature/mature B cells from the BM, pre- and pro-B cells appear to be the main source of myeloid-B cells in the BM as reported elsewhere (89).

Considering the role of GM-CSF in myelopoiesis (90), we assessed the expression of GM-CSF receptor (R) in CD19+ cells in the BM of infected mice. We found a significant increase in GM-CSFR expression in CD19+ cells in the BM of infected mice (Fig. 7J and K). Thus, we hypothesized whether increased GM-CSFR results in the expansion of CD11b+ B cells in the BM of infected mice. To test our hypothesis, we treated BM cells with rGM-CSF (10 ng/mL) for 48 hr, which showed a significant increase in percentages of Ly6G + Ly6C + myeloid cells within the CD19+ cell subset from infected compared with control mice (Fig. 7L and M). Given the essential role of IL-7 in B cell development and maintenance (89), we investigated the effects of rIL-7 and GM-CSF on the frequency of BM CD19 + B220+ cells within the CD11b+ subset. As anticipated, rIL-7 significantly increased, but rGM-CSF decreased the proportion of CD19+ B220+ cells from BM cells of infected mice *in vitro* (Fig. 7N and O). To confirm this observation, we treated mice with rIL-7 (5 µg/mouse) and anti-GM-CSF (100 µg/mouse) for 2 consecutive days post-infection. We found that this treatment maintained the proportion of CD19+ CD220+ cells in the BM of infected mice (Fig. 7P and Q) and prevented their conversion to CD11b+ cells (Fig. 7R), whereas single treatment with either rIL-7 or anti-GM-CSF did not significantly affect the proportion of CD19+ CD220+ cells (Fig. S5F). Despite a previous report (87), we did not observe any changes in the frequency of CD19+ B220+ cells once BM cells were treated with rIL-10 for 48 hr *in vitro*. This discrepancy might be related to using different models (LPS vs infection) or *in vivo* vs *in vitro* studies. These observations suggest that acute severe infection results in the expansion of myeloid cells at the expense of a contraction in B cell generation.

### Reduction of B cells within the Gr1+ cells in the BM following infection

**Fig 8:**
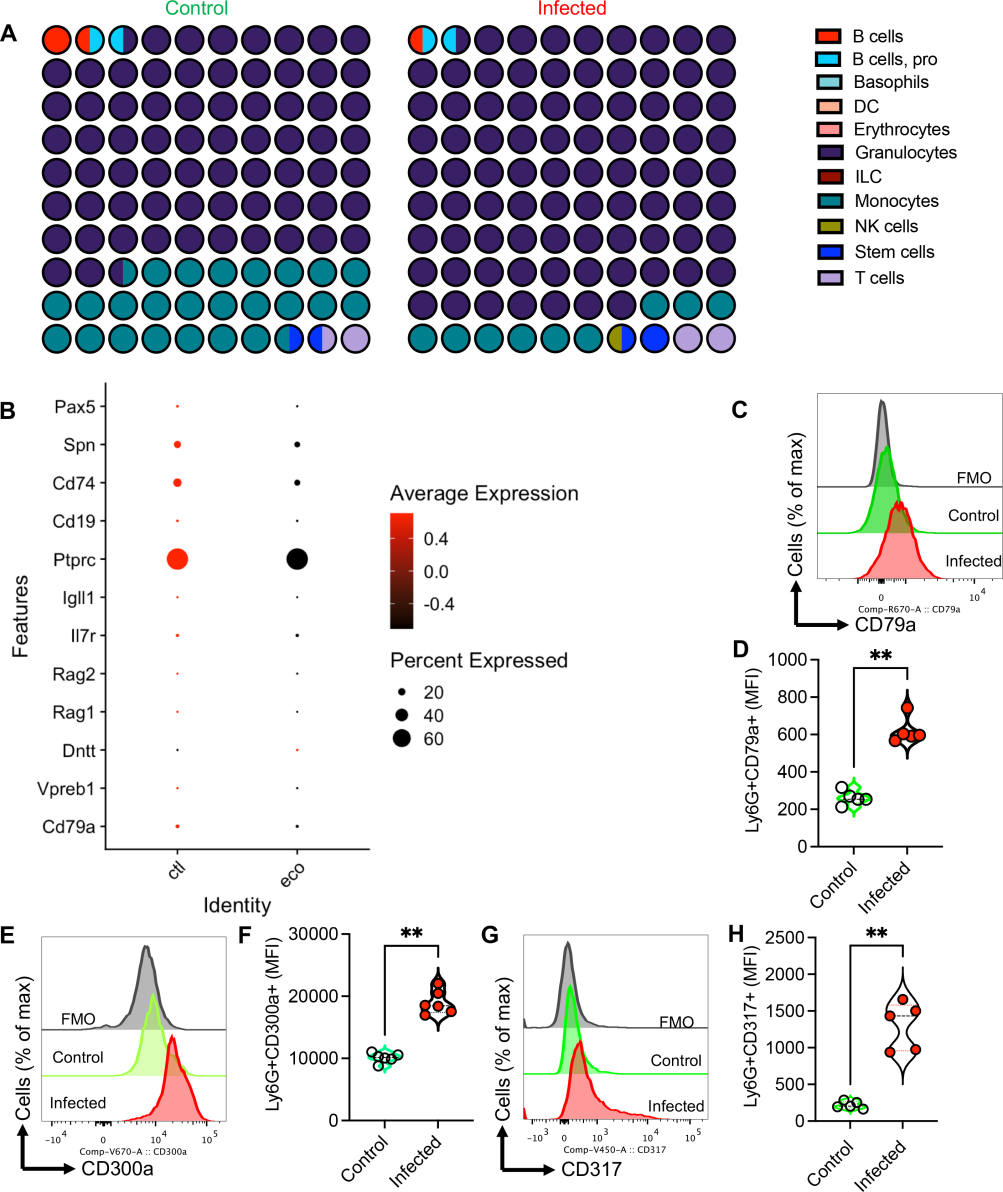
A reduction in B cell and B cell-associated genes in the BM of infected mice. (**A**) Fraction plots based on scRNAseq analysis showing the percentage of genes associated with different immune cells in the bone marrow of a control and an infected mouse with *E. coli*. (**B**) Bubble plot of differentially expressed genes associated with B cell lineage genes in Gr-1+ cells from the BM of a control and an infected mouse with *E. coli*. (**C**) Representative flow cytometry plots and (**D**) cumulative data of CD79a expression in Ly6G+ cells in the BM of control and infected mice. (**E**) Representative flow cytometry plots and (**F**) cumulative data of CD300a expression in Ly6G+ cells in the BM of control and infected mice. (**G**) Representative flow cytometry plots and (**H**) cumulative data of CD317 expression in Ly6G+ cells in the BM of control and infected mice. Each dot represents data from an animal either control or infected. *P* values were calculated using two-tailed, Mann-Whitney *t* test (**D,F,H**). **, *P* < 0.01. Fluorescence minus one (FMO). Control (ctl) and E. coli-infected (eco).

The publicly available scRNAseq data set (47) was reanalyzed to determine the pattern of B cells within granulocytes in the BM of mice following infection with *E. coli*. In consistent with our *in vivo* studies, these analyses revealed a substantial reduction in B cells within the granulocyte subset (Gr1+ cells) in the BM of infected mice (Fig. 8A; Fig. S6). This was further confirmed when we found a reduction in Pro-Pre-associated B cell genes in Gr1+ cells in the BM of infected mice (Fig. 8B). Moreover, we randomly selected a few genes based on the availability of their markers and assessed their presence at the protein level in control versus infected mice. These studies revealed that CD79a, CD300a, and CD317 associated with Pre-Pro B cells were significantly elevated at the protein levels in Ly6G+ T cells in infected mice compared with the control group (Fig. 8C through E). These observations support the hypothesis that stress hematopoiesis following infection skews Pro-Pre-B cells toward myeloid cells (e.g., Ly6G cells).

### A lower percentage of B cells in PBMCs of COVID-19 patients

**Fig 9:**
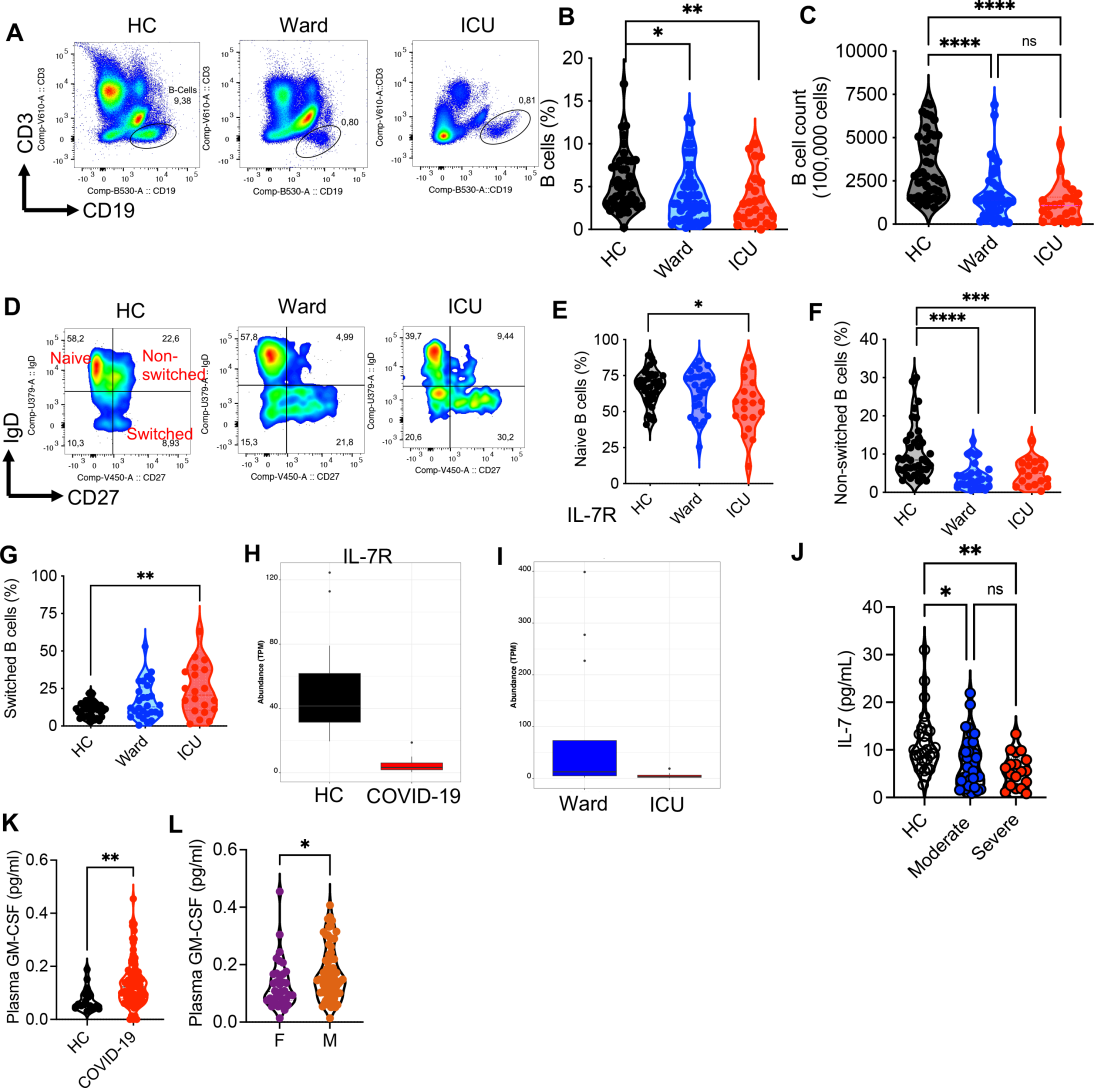
Lower frequency of B cells in the PBMCs of COVID-19 patients. (**A**) Representative flow cytometry plots, and cumulative data showing (**B**) percentages and (**C**) absolute count of B cells out of 100,000 PBMCs in different groups. (**D**) Representative flow cytometry plots, and cumulative data showing percentages of (**E**) naïve, (**F**) non-switched, and (**G**) switched B cell subsets in different groups. (**H**) Abundance of IL-7R transcripts as shown by transcripts per million in neutrophils from HCs vs COVID-19 patients, and (**I**) in neutrophils from ward-admitted vs ICU-admitted COVID-19 patients. (**J**) IL-7 levels in the plasma of HC vs COVID-19 patients either admitted to ward or ICU. (**K**) GM-CSF levels in the plasma of HC vs COVID-19 patients, and (**L**) in female (**F**) vs male (**M**) COVID-19 ICU-admitted patients. Each dot represents data from a human study subject. *P* values were calculated using two-tailed, Mann-Whitney *t* test (**H,I, K,L**). One-way ANOVA (**B,C,E,F,G,J**). *, *P* < 0.05, **P < 0.01, ***, *P* < 0.001, and ****, and *P* < 0.0001. Health control (HC), COVID-19 patients admitted to hospital ward (ward/moderate) or intensive care unit (ICU/severe). Female (**F**) and male (**M**).

Since our results support the concept of B cell progenitors’ conversion to myeloid cells in stress hematopoiesis (e.g., infection), we quantified the percentages of B cells in PBMCs of our COVID-19 patients. These observations showed a significantly lower percentage and absolute number of B cells in PBMCs of COVID-19 patients compared with HCs (Fig. 9A through C) as reported elsewhere (91). We further found a lower frequency of naïve and non-switched B cell subsets but increased percentages of switched B cell subsets in ICU-admitted patients compared with HCs (Fig. 9D through G). Of note, COVID-19 patients in hospital wards also exhibited a lower percentage of non-switched B cells compared with HCs (Fig. 9F).

Considering our observations in the animal infection model, we questioned whether a differential in the expression level of GM-CSFR/IL-7R in neutrophils of COVID-19 patients or the plasma concentrations of GM-CSF and/or IL-7 may explain the reduction in the B cell population. We found a significant reduction in IL-7R in neutrophils from COVID-19 patients compared with HCs (Fig. 9H) and ward versus ICU-admitted patients (Fig. 9I). Likewise, a significant reduction in IL-7 levels in the plasma of COVID-19 patients either admitted to the ICU or ward compared with HCs was noted (Fig. 9J). Although we did not observe any increase in GM-CSFR in neutrophils from COVID-19 patients, we noted an elevation in the plasma GM-CSF in COVID-19 patients (Fig. 9K) as reported elsewhere (34). It is worth mentioning that GM-CSF was significantly higher in the plasma of male than female ICU-admitted patients (Fig. 9L). These findings suggest a potential role for the elevation of GM-CSF but the decline of IL-7 in the promotion of myeloid cells in COVID-19 infection. However, further studies are needed to support this hypothesis in humans.

## DISCUSSION

The innate immune system as the evolutionary first line of defense rapidly recruits immune cells to the site of infection/inflammation. The major players of this system such as neutrophils and monocytes are generated in the BM under steady immune hemostasis. However, in response to systemic infection, a large number of myeloid cells are generated via emergency myelopoiesis. This skewed myelopoiesis is essential to rapidly eliminate invading pathogens. The existence of a small subset of bipotential B-macrophage within the BM of mice under steady state supports this notion (92). Importantly, in the context of systemic infection, the enforcement of myelopoiesis from B cell lineages within the BM of mice serves as an additional endorsement for the role of a distinct B cell subset in facilitating emergency myelopoiesis (87). However, the role of lymphoid cells in this process has not been well understood in humans. In this study, we investigated the impact of SARS-CoV-2 infection on neutrophils in those with moderate (ward) or severe (ICU) disease. Our study revealed distinct transcriptional profiles of neutrophils from ward-admitted and ICD-admitted COVID-19 patients once compared with HCs. Likewise, we found that ICU-admitted patients displayed substantially more up- and down-regulated genes than those in the ward, indicating more pronounced transcriptional alterations in severe COVID-19 disease. Notably, we observed several genes associated with chemotaxis, cytotoxicity, and phagocytosis were upregulated in neutrophils from COVID-19 patients, especially those in the ICU, which is in line with another report (82). Of note, we found a differential transcriptional profile for neutrophils in ward-admitted vs ICU-admitted male but not female patients. The difference between female and male neutrophils has already been documented. For example, neutrophils in adult males appear to display enrichment of immature genes (93). However, neutrophils in females exhibit hyperresponsiveness to interferon (IFN), resulting in upregulation of the type I IFN pathway (78). Considering that emergency hematopoiesis is associated with the emergence of neutrophil progenitors/precursors in the circulation (1), our finding demonstrated the abundance of these young neutrophils, particularly in those admitted to the ICU. An intriguing observation was the enrichments of Ig genes and B cell lineage-associated genes in neutrophils from SARS-CoV-2-infected individuals, which was more prominent in those admitted to the ICU. The presence of B cell lineage-associated genes in neutrophils implies potential lineage plasticity or hybrid cell phenotype under the influence of emergency myelopoiesis (e.g., severe systemic infection). This observation provides a novel insight into the suppression of B but not T lymphocytes during emergency myelopoiesis. The appearance of B-cell-associated genes in neutrophils of COVID-19 patients suggests a complex interplay between different immune cell lineages in response to severe infection. Moreover, our finding raises questions about the regulatory mechanisms and potential roles of these hybrid cells in the context of SARS-CoV-2 or other infections. The highest expression of Ig and B cell lineage-associated genes in neutrophils of ICU compared with ward patients suggests a greater expansion of bipotential B-neutrophils in COVID-19 patients with more severe disease. We validated our RNAseq findings in a much larger cohort of ICU-admitted COVID-19 patients (82). Moreover, reanalyzing of a scRNAseq data set confirmed the presence of hybrid cells in human BM under steady physiological conditions, which is in agreement with another report (94). In particular, we confirmed the existence of B cell-associated markers, such as IgM, CD19, and CD24 at the protein levels in granulocytes from the BM of human subjects. These findings support the presence of bipotential B-neutrophils in human BM (83) that can be skewed toward neutrophils upon emergency myelopoiesis. While neutrophils as the first line of defense may play a crucial role in the clearance of invading pathogens, emergency myelopoiesis is associated with severe COVID-19 disease (95). It is associated with poor clinical outcomes and characterized by progressive lymphopenia and anemia (95) that may also reflect relative immunoparesis, a phenomenon associated with increased morbidity and mortality, notably in the anti-inflammatory phase of sepsis (96). In contrast, the genes associated with T and NK cells were downregulated in neutrophils from COVID-19 patients. However, we found the enrichment of erythrocyte and monocyte-associated genes in neutrophils of COVID-19 patients. Since neutrophils, erythrocytes, and monocytes are derived from the CMPs, the presence of gene signature of erythrocytes and monocytes in neutrophils of COVID-19 patients reflects the egress of CMPs from the BM under emergency myelopoiesis. It is worth mentioning that SARS-CoV-2 impacts erythropoiesis as evidenced by the abundance of erythroid progenitors and precursors in the peripheral blood of COVID-19 patients (97, 98). However, the observation of B cell signature genes that originate from CLPs in the early stages of maturation implies the presence of hybrid progenitor cells in the BM with the potential to generate both neutrophils and B cells. This phenomenon may explain altered immune responses and potential dysregulated/impaired B cell development in COVID-19 patients. In support of our observations, the presence of neutrophils with transcriptional and surface markers reminiscent of B cells has been reported in the peripheral blood of patients with early-stage melanoma (94).

We confirmed this phenomenon in animal studies, where a substantial reduction in total B cells and their associated transcripts (e.g., Pre-Pro B cells) was noted in Gr1+ cells in the BM of infected mice with *E. coli*. (47) Furthermore, we observed a significant increase in the expression of B cell-associated proteins such as CD79a, PST2 (CD317), and CD300a in Ly6G+ cells in infected mice with *E. coli*. These observations suggest the presence of a well-tuned interplay by which B cells promote the on-demand production of neutrophils as the first line of defense to combat invading pathogens.

In this context, we suggest a potential role for GM-CSF and IL-7 in modulating B and neutrophil dynamics during severe systemic infection (e.g., SARS-CoV-2) and bacterial infections. Therefore, increased levels of GM-CSF receptor and elevated GM-CSF levels in the plasma may contribute to the expansion of myeloid cells (99), including neutrophils, at the expense of B cell generation. Conversely, reduced IL-7 receptor expression in neutrophils and lower IL-7 levels in the plasma of COVID-19 patients (31) could possibly impact B cell development. This hypothesis was further supported when the treatment of infected mice with the anti-GM-CSF blocking antibody and rIL-7 abrogated the diversion of B cells to neutrophils. These results indicate that a reduction in IL-7 but an increase in GM-CSF may trigger myeloid-biased hematopoiesis in the BM. Taken together, our results offer insights into the complex immune response to severe systemic infection and stress hematopoiesis and their impact on neutrophils and B cell development. The observed differences in neutrophil transcriptional profiles and the concept of bipotential B-neutrophil existence could contribute to our understanding of severe infection pathogenesis. It is worth mentioning that our results refute the claim that a high expression of the Ig genes in neutrophils of COVID-19 patients was deemed to be associated with B cell contamination (82). The authors have utilized CIBERSORTx cell type fractions to detect non-neutrophil contamination, which uses the gene signatures to define the cell type. The CIBERSORTx cell type reported the existence of B cell contamination based on the high presence of Ig and other B cell-related genes in neutrophils of COVID-19 patients. Therefore, we believe the high expression of Ig and B cell-associated genes in neutrophils of this cohort was related to the presence of hybrid cells that express both B cell and neutrophil genes.

We are aware of multiple study limitations such as the need for further functional validations and the potential role of other factors influencing B-neutrophil gene expression. Another limitation of our study is the inability to explore the physiological relevance and role of these B-neutrophils in pathological conditions. However, a recent study has reported that CD79b+ neutrophils in tumors are primed for NETosis and display greater phagocytic capacity (94). Therefore, we believe that these hybrid cells may play an important role in pathological conditions that need further exploration. We were also limited by the inability to use an animal viral infection instead of a bacterial infection model (e.g., *E. coli*). Therefore, investigating B-neutrophil induction in animal viral infection models is warranted. We acknowledge that neutrophils are highly heterogeneous. A recent scRNAseq has revealed eight neutrophil subsets by distinct molecular signatures in the BM of mice (47). Similarly, extreme diversity in human neutrophils has been reported under stress myelopoisis (100). Moreover, granulocytic myeloid-derived suppressor cells (G-MDSCs) with distinctive immunosuppressive properties are also generated under stress hematopoiesis, cancer, and other pathological conditions (101, 102). Therefore, further studies are needed to appreciate the heterogeneity of neutrophil subpopulations and determine the presence of B-neutrophils in different subsets. It is worth mentioning that MDSCs, as light-weight neutrophils, are in the buffy coat layer upon blood processing, while we performed our RNAseq-related studies on heavyweight neutrophils (24).

It is worth mentioning that our human study subjects were infected with the Wuhan strain of SARS-CoV-2. Considering the variability of viral pathogenesis (103), whether the same effects can be observed by other SARS-CoV-2 variants of concern and long-COVID patients (104) remains to be determined.

In conclusion, our study sheds light on the differential transcriptional profile of neutrophils from COVID-19 patients and highlights potential cross-talk between B cell and neutrophil lineages during severe infection and stress hematopoiesis. Our findings have implications for the understanding of immune response in stress hematopoiesis, and further studies may provide valuable insights into the role of bipotential B-neutrophils in disease progression and potential therapeutic interventions.

## Data Availability

All the generated data related to this study are incorporated in the main and supplemental figures. The original data related to RNAseq are available from the SRA portal on NCBI under Accession Number PRJNA671810. The other publicly available data sets due to their relevance to our study were analyzed that are available from the GEO repositories: GSE117498, GSE212041, GSE137539.

## SUPPLEMENTAL MATERIAL

Supplemental figures mbio.01599-24-s0001.pdf Figures S1 to S6.

ASM does not own the copyrights to Supplemental Material that may be linked to, or accessed through, an article. The authors have granted ASM a non-exclusive, world-wide license to publish the Supplemental Material files. Please contact the corresponding author directly for reuse.
