# Supplementary material for: Bipotential B-neutrophil progenitors are present in human and mouse bone marrow and emerge in the periphery upon stress hematopoiesis
Source: mBio. 2024 Jul 16;15(8):e01599-24. doi: 10.1128/mbio.01599-24 (PMC11323571; doi:10.1128/mbio.01599-24)
Supplement: Supplemental figures — Figures S1 to S6. [file mbio.01599-24-s0001.pdf]

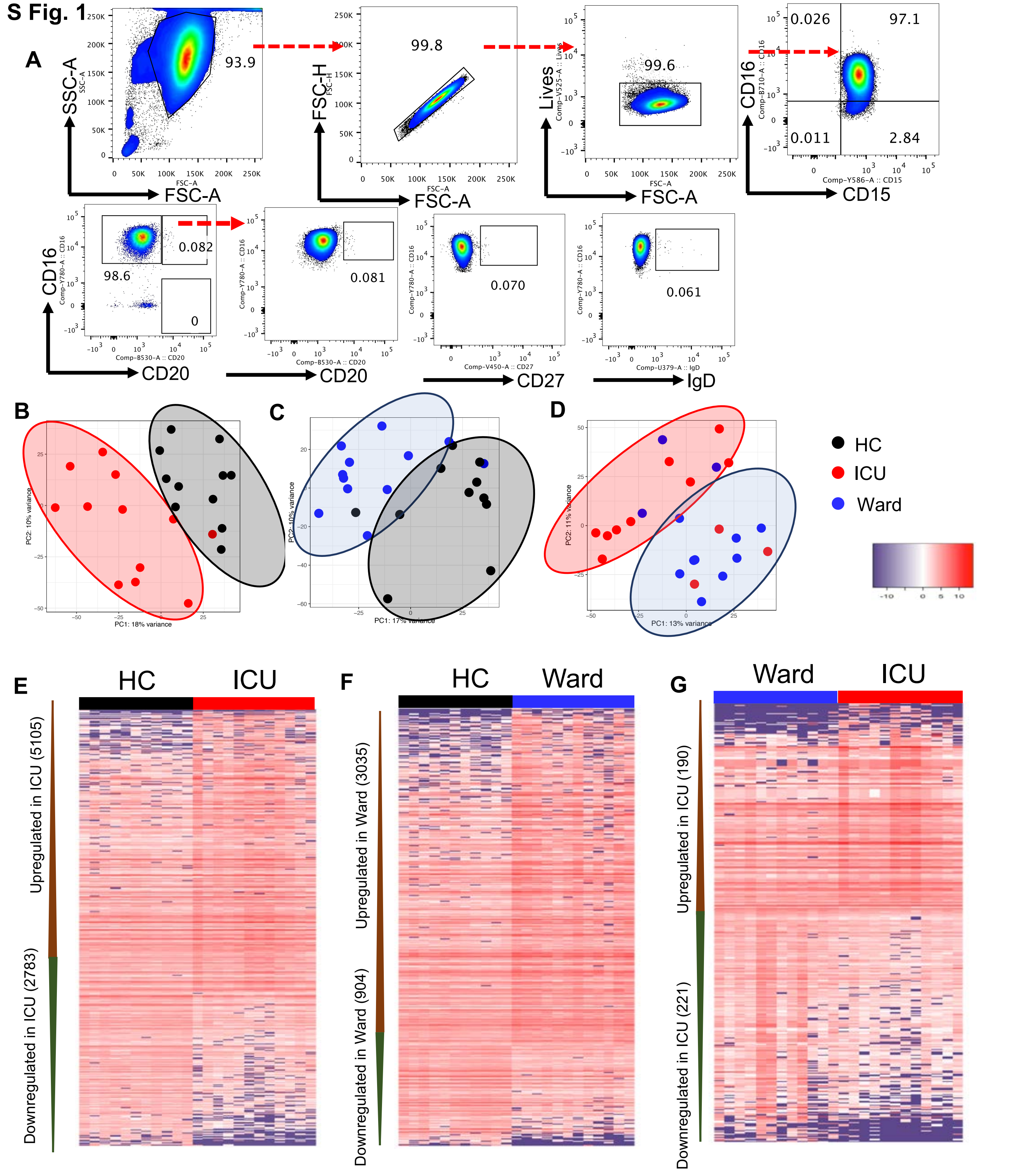

S Fig. 1
A
SSC-A
FSC-A
93.9
FSC-H
99.8
Lives
99.6
CD16
CD15
0.026
97.1
0.011
2.84
98.6
0.082
0
CD20
0.081
CD27
0.070
IgD
0.061
B
C
D
PC1: 18% variance
PC2: 10% variance
PC1: 17% variance
PC1: 13% variance
PC2: 11% variance
HC
ICU
Ward
E
F
G
Upregulated in ICU (5105)
Downregulated in ICU (2783)
Upregulated in Ward (3035)
Downregulated in Ward (904)
Upregulated in ICU (190)
Downregulated in ICU (221)

S Fig. 2

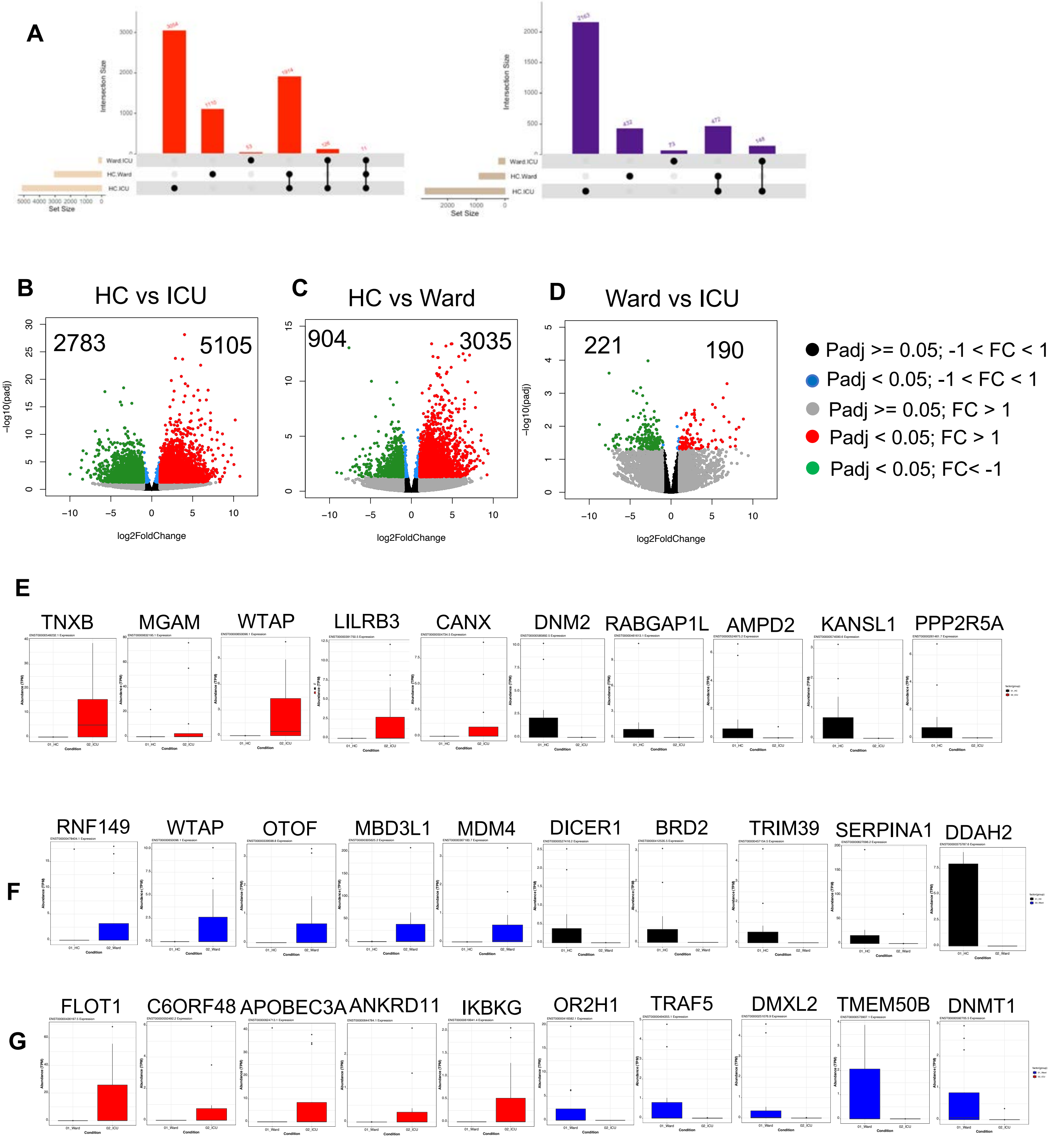
A
B
HC vs ICU
2783
5105
C
HC vs Ward
904
3035
D
Ward vs ICU
221
190
Padj >= 0.05; -1 < FC < 1
Padj < 0.05; -1 < FC < 1
Padj >= 0.05; FC > 1
Padj < 0.05; FC > 1
Padj < 0.05; FC< -1
log2FoldChange
-log10(padj)
E
TNXB
MGAM
WTAP
LILRB3
CANX
DNM2
RABGAP1L
AMPD2
KANSL1
PPP2R5A
F
RNF149
WTAP
OTOF
MBD3L1
MDM4
DICER1
BRD2
TRIM39
SERPINA1
DDAH2
G
FLOT1
C6ORF48
APOBEC3A
ANKRD11
IKBKG
OR2H1
TRAF5
DMXL2
TMEM50B
DNMT1

S Fig. 3

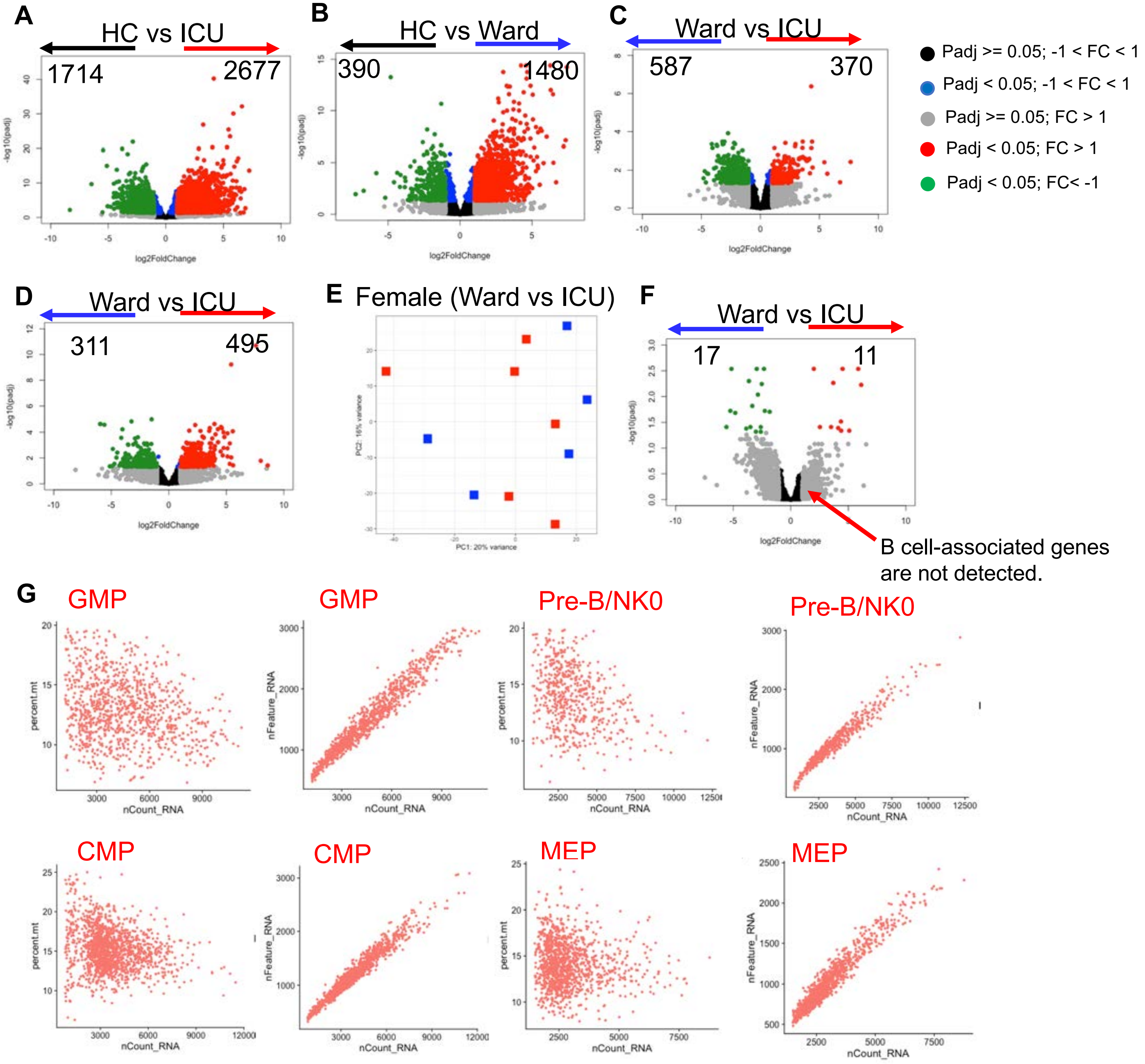
A
HC vs ICU
1714
2677
B
HC vs Ward
390
1480
C
Ward vs ICU
587
370
Padj >= 0.05; -1 < FC < 1
Padj < 0.05; -1 < FC < 1
Padj >= 0.05; FC > 1
Padj < 0.05; FC > 1
Padj < 0.05; FC< -1
D
Ward vs ICU
311
495
E
Female (Ward vs ICU)
F
Ward vs ICU
17
11
B cell-associated genes are not detected.
G
GMP
GMP
Pre-B/NK0
Pre-B/NK0
CMP
CMP
MEP
MEP
log2FoldChange
-log10(padj)
percent.mt
nFeature_RNA
nCount_RNA

S Fig. 4

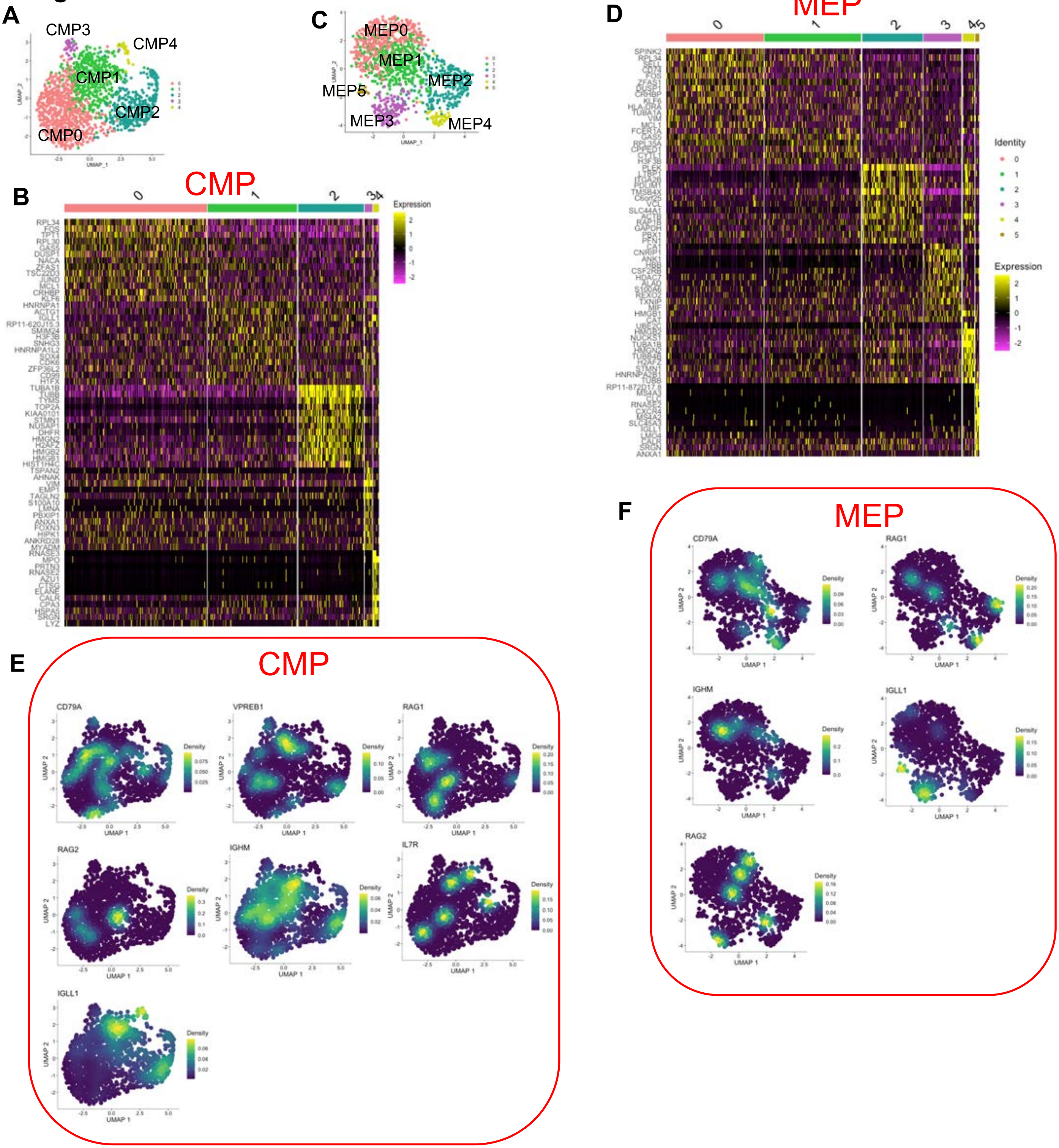
A
CMP3
CMP4
CMP1
CMP2
CMP0
UMAP_1
UMAP_2
C
MEP0
MEP1
MEP2
MEP5
MEP3
MEP4
UMAP_1
UMAP_2
D
MEP
Identity
Expression
B
CMP
Expression
E
CMP
CD79A
VPREB1
RAG1
RAG2
IGHM
IL7R
IGLL1
Density
UMAP 1
UMAP 2
F
MEP
CD79A
RAG1
IGHM
IGLL1
RAG2
Density
UMAP 1
UMAP 2

S Fig. 5

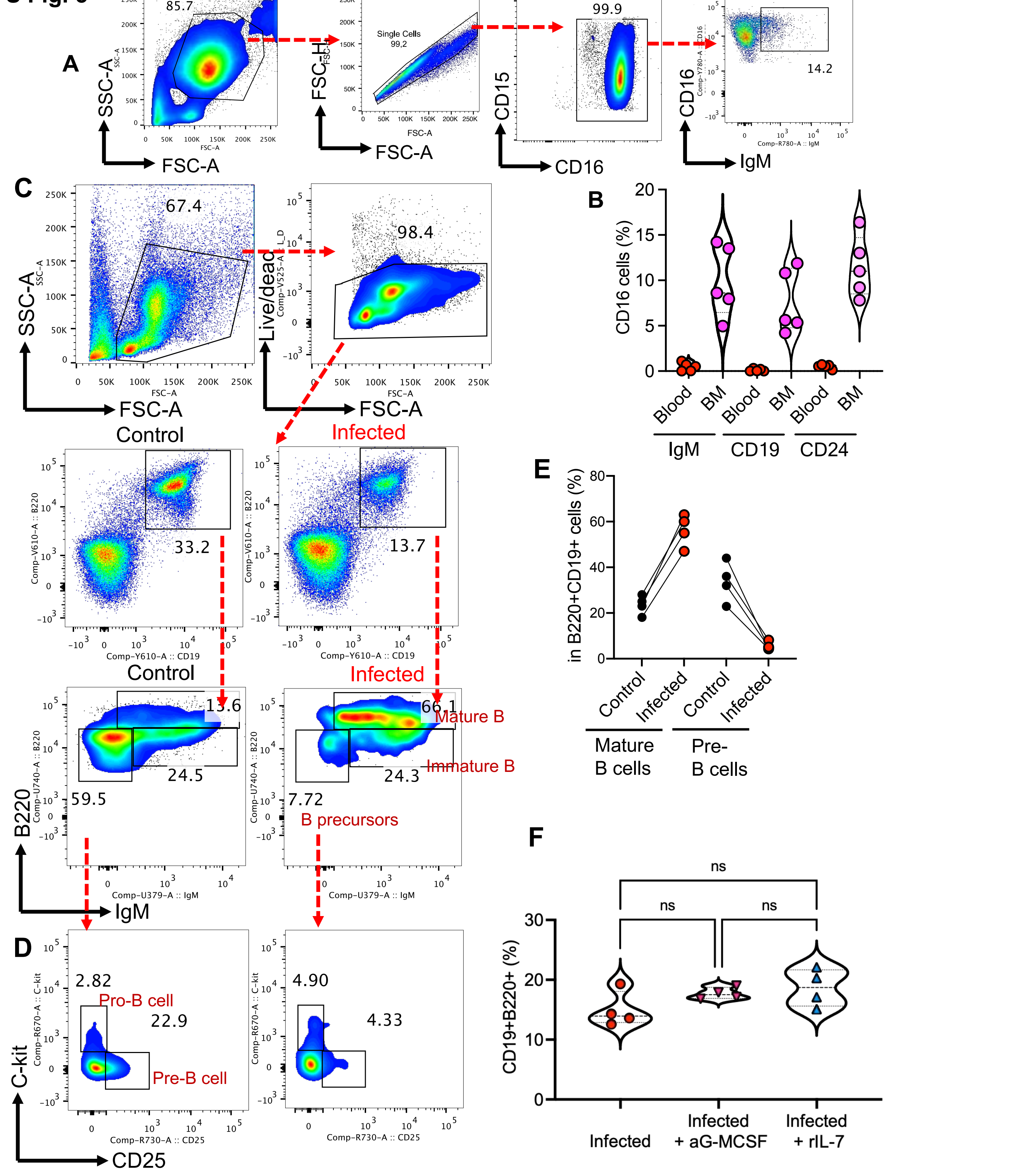
A
SSC-A
FSC-A
85.7
FSC-H
Single Cells
99,2
99.9
CD15
CD16
14.2
IgM
B
CD16 cells (%)
Blood
BM
IgM
CD19
CD24
C
67.4
Live/dead
98.4
Control
Infected
33.2
13.7
Comp-Y610-A :: CD19
Comp-V610-A :: B220
13.6
66.1
Mature B
24.5
24.3
Immature B
59.5
7.72
B precursors
B220
Comp-U379-A :: IgM
D
2.82
Pro-B cell
22.9
Pre-B cell
4.90
4.33
C-kit
CD25
E
in B220+CD19+ cells (%)
Control
Infected
Mature B cells
Pre- B cells
F
ns
CD19+B220+ (%)
Infected
Infected + aG-MCSF
Infected + rIL-7

S Fig. 6

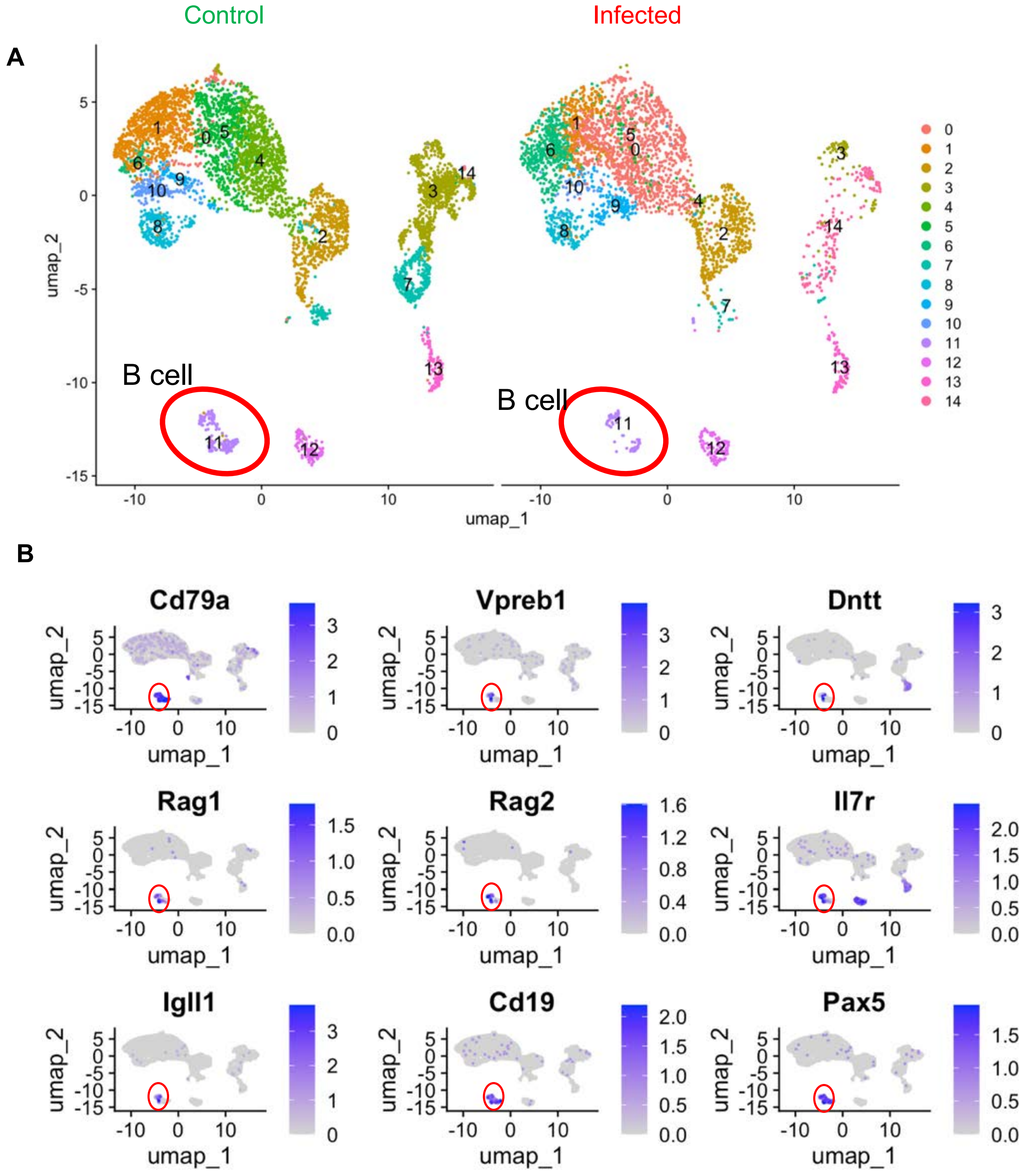
A
Control
Infected
B cell
B cell
umap_1
umap_2
0
1
2
3
4
5
6
7
8
9
10
11
12
13
14
B
Cd79a
Vpreb1
Dntt
Rag1
Rag2
Il7r
Igll1
Cd19
Pax5

Supplemental figure legends

S Fig. 1. (**A**) Representative flow cytometry plots showing the gating strategy and purity of isolated neutrophils for RNAseq. (**B-D**) PCA on the Euclidian distances of the transcripts between neutrophils of different study cohorts as indicated. Heat maps describing normalised abundance of differentially expressed transcripts between (**E**) HC and ICU, (**F**) HC and ward-admitted, and (**G**) ward and ICU-admitted COVID-19 patients. Regularised logarithmic transformation was applied to the raw counts prior to heat map plotting. Only transcripts with $P_{\text{adj}} < 0.05$ and a $\log_2 -1 <$ fold change (FC) $> +1$ are included. The number of upregulated or downregulated transcripts in each comparison is indicated with brown or green vertical bars on the left of each heat map and included in parentheses.

S Fig. 2. (**A**) UpSet plots depicting upregulated (Red) and downregulated (blue) transcripts in neutrophils as shown differentially expressed (DE) in one or more comparisons. Sidebars (pinkish-brown bars) represent the number of transcripts DE in each comparison. Red and purple vertical bars represent the intersection size, which is the number of transcripts that were up- and down-regulated in one or more comparisons, respectively. Black dots represent comparisons in which transcripts were found DE. For instance, the left-most vertical bar in each panel indicates that 3054 and 2163 transcripts, respectively, were up- or downregulated only when comparing neutrophils from ICU-admitted vs HC. Black dots connected with a line indicate transcripts DE in more than one comparison. Volcano plots depicting the number and FC of differentially expressed transcripts in neutrophils of (**B**) HCs vs ICU, (**C**) HCs vs ward, and (**D**) ward vs ICU groups. Black dots represent transcripts that were not differentially expressed between different groups. Grey dots represent transcripts that were not differentially expressed between different groups but with a -1 < FC < 1. Red dots represent DE transcripts with a FC > 1. Green dots represent DE transcripts with a FC < -1. Blue dots represent DE transcripts with a -1 < FC < 1. The numbers of DE up- and down-regulated transcripts were noted in the right and left sides of each plot, respectively. (**E**) Box plots showing the abundance or transcript per million (TPM) of the 5 most upregulated and downregulated transcripts in neutrophils of HC vs ICU. (**F**) Box plots showing the TPM of 5 most upregulates and downregulated transcripts in neutrophils of HC vs ward-admitted patients. (**G**) Box plots showing the TPM of 5 most upregulates and downregulated transcripts in neutrophils of ward vs ICU-admitted COVID-19 patients.

S Fig. 3. Volcano plots depicting the number and FC of differentially expressed genes in neutrophils of (**A**) HCs vs ICU-admitted, (**B**) HCs vs ward-admitted, and (**C**) ward vs ICU-admitted patients. (**D**) Volcano plots depicting the number and FC of differentially expressed genes in neutrophils of male patients admitted to ward or ICU. (**E**) PCA on the Euclidian distances of the genes between neutrophils of female patients admitted to ward or ICU. (**F**) Volcano plots depicting the number and FC of differentially expressed genes in neutrophils from female patients admitted to ward vs ICU. Black dots represent transcripts that were not differentially expressed between different groups. Grey dots represent transcripts that were not differentially expressed between different groups but with a -1 < FC < 1. Red dots represent DE transcripts with a FC > 1. Green dots represent DE transcripts with a FC < -1. Blue dots represent DE transcripts with a -1 < FC < 1. The numbers of DE up- and down-regulated transcripts were noted in the right and left sides of each plot, respectively. (**G**) Quality control of scRNAseq data for each library of GMP, Pre-B/NK0, CMP, and MEP subpopulations was performed by plotting the percent of mitochondrial genes and the number of genes against total number of molecules present in each cell in thousands (nCount).

S Fig. 4. (**A**) UMAP plot of CMP clusters. (**B**) The top 13 up-regulated genes in every CPM cluster. (**C**) UMAP plot of MEP clusters. (**D**) The top 20 up-regulated genes in every MEP cluster. (**E**) Density plots depicting the level of expression of B-cell linage associated genes in CMP and (**F**) MEP subpopulations.

S Fig. 5. (**A**) Representative flow cytometry showing the gating strategy for neutrophils and IgM expression in BM aspirates of human subjects. (**B**) Cumulative data for the expression of IgM, CD19, and CD24 in neutrophils from BM aspirates of human subjects. (**C**) The gating strategy for different B cell subsets in BM of either control or infected mice (48 hr post infection). (**D**) Representative plots,

and (**E**) cumulative data of % mature and pre-B cells in controls vs infected mice. (**F**) Cumulative data of the percentages of CD19+B220+ cells in the BM of infected mice versus those either treated with anti-G-MCSF (aG-MCSF @ 100 μg/mouse) or recombinant IL-7 (rIL-7 @ 5 μg/mouse). Not significant (ns).

S Fig. 6. (**A**) UMAP plot of Gr-1+ cells isolated from the BM of control and infected mice with *E. coli*. (**B**) Featured plots showing the expression of B-cell linage associated genes in Gr-1+ cells from the BM of mice.
